# Metasurface‐Driven Adaptive Structured Light: Achieving Integrated Real‐Time 3D Reconstruction and Forward Ranging in Multi‐Scene

**DOI:** 10.1002/advs.202507339

**Published:** 2025-07-14

**Authors:** Zhengren Zhang, Mengran Yang, Anjun Qu, Zile Li, Shaohua Yu, Guoxing Zheng

**Affiliations:** ^1^ School of Materials Science and Engineering Chongqing Jiaotong University Chongqing 400074 China; ^2^ Peng Cheng Laboratory Shenzhen 518055 China; ^3^ Wuhan Institute of Quantum Technology Wuhan 430206 China; ^4^ Electronic Information School, and School of Microelectronics Wuhan University Wuhan 430072 China

**Keywords:** 3D reconstruction, adaptive, forward ranging, metasurface, real‐time, structured light

## Abstract

In structured light‐based three‐dimensional (3D) reconstruction technology, normal and grazing emission beams hold significant application value due to their stable axial propagation characteristics and anti‐distortion advantages. However, current systems face two major technical bottlenecks. First, traditional grazing emission beams depend on the precise assembly of complex optical components, which compromises reliability and impedes miniaturization. Second, the physical constraints of light field transmission make it challenging to generate both modes simultaneously within a single system. This study exploits the light waves modulation capabilities of metasurface to achieve the simultaneous generation of normal and grazing emission dual‐mode beams. Specifically, an adaptive closed‐line structured light with a central bright spot is generated within 2π space. By integrating a self‐developed multimodal point cloud enhancement algorithm, a 3D reconstruction and forward ranging system adaptable to diverse scenarios has been successfully developed. This system achieves real‐time 3D reconstruction and forward ranging at 30 fps without additional ranging modules. This technology not only transforms the hardware architecture of existing structured light systems and surpasses the limit of light emission angles but also overcomes the application constraints of traditional methods in diverse scenarios. It provides innovative technological support and solutions for the digital construction of tunnel engineering.

## Graphical abstract

A metasurface‐based structured light system for real‐time three‐dimensional reconstruction and forward ranging is demonstrated. By simultaneously emitting grazing and normal emission beams, hardware limitations are overcome. Combining adaptive closed‐line light patterns with a point cloud enhancement algorithm enables 30 frames per second imaging in tunnels, achieving miniaturized, high‐performance depth sensing without additional ranging modules.

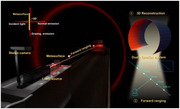

## 1 Introduction

Normal emission and grazing emission are two typical propagation modes of emitted beams. Normal emission beams propagate vertically to the medium interface, with their wavefront phase gradient approaching zero, allowing for stable axial propagation. In contrast, grazing emission beams exhibit nearly parallel propagation to the interface and require high phase gradients to achieve large‐angle deflection (close to 90°). The difference in physical properties gives the two modes complementary advantages in the design of optical three‐dimensional (3D) reconstruction systems.^[^ ^1^, ^2^, ^3^ ^]^ Grazing emission not only enables ultra‐wide field structured light projection but also minimizes structured light distortion, thereby reducing the computational burden on algorithms. This makes it particularly suitable for large‐scale 3D reconstruction. On the other hand, normal emission can generate high‐intensity axial light spots, making it suitable for distance measurement. However, current diffractive optical elements (DOEs) are constrained by pixel period limitations, making it difficult for beams to cover large areas (see Section S1, Supporting Information). Consequently, traditional structured light systems rely on the precise integration of microelectromechanical systems (MEMS) or prisms to achieve grazing emission, which negatively impacts the reliability and miniaturization of 3D reconstruction systems.^[^ ^4^, ^5^ ^]^ Furthermore, to reduce the interference of normal emission light on the field near the optical axis, the additional optical components used to suppress the zero‐order diffraction will increase system complexity. These factors lead to the fact that, although normal emission and grazing emission have complementary advantages, they are typically not used simultaneously. If both grazing emission and normal emission can be utilized together, it would significantly improve light path efficiency, reduce system complexity, and promote system integration.

Metasurfaces are a 2D material with unit structures smaller than the wavelength, enabling flexible and efficient control over electromagnetic wave properties, including polarization, amplitude, phase, and propagation modes. It is considered critical for breaking through the limitations of traditional optical technologies. Leveraging their outstanding electromagnetic wave control capabilities,^[^ ^6^, ^7^, ^8^, ^9^, ^10^, ^11^, ^12^, ^13^ ^]^ metasurfaces have found widespread applications in optical fields, including holographic displays^[^ ^14^, ^15^, ^16^, ^17^ ^]^ and beam shaping.^[^ ^18^, ^19^, ^20^ ^]^ The features of miniaturization, easy integration, and full‐space tunability have unlocked unprecedented potential for low‐power, compact systems and 3D imaging in wide field‐of‐view scenes.^[^ ^21^, ^22^ ^]^ Specifically, metasurfaces can precisely control the phase of incident light to generate grazing emission structured light with an angle close to 90°,^[^ ^23^, ^24^, ^25^ ^]^ while normal emission generated simultaneously creates a strong central bright spot on the illuminated object, ideal for forward ranging. Therefore, metasurfaces can achieve both grazing and normal emission with a single ultrathin‐element, fulfilling the dual needs of large‐scale 3D reconstruction and forward ranging, offering an excellent platform for constructing a new 3D reconstruction system that integrates both functions. Compared with Light Detection and Ranging^[^ ^26^, ^27^ ^]^ (LiDAR), photogrammetry,^[^ ^28^, ^29^ ^]^ and structured light techniques,^[^ ^30^, ^31^, ^32^ ^]^ the proposed 3D reconstruction system offers significant advantages in hardware maintenance costs, data collection complexity, and integration. Additionally, DOEs used in traditional structured light systems have limited projection capabilities in large‐scale scenarios, which can lead to spot distortion and impact the 3D reconstruction results.^[^ ^33^, ^34^ ^]^ Grazing emission structured light can reduce spot distortion, decrease computational burden, and improve the system's applicability in multiple scenarios.

**Figure 1:**
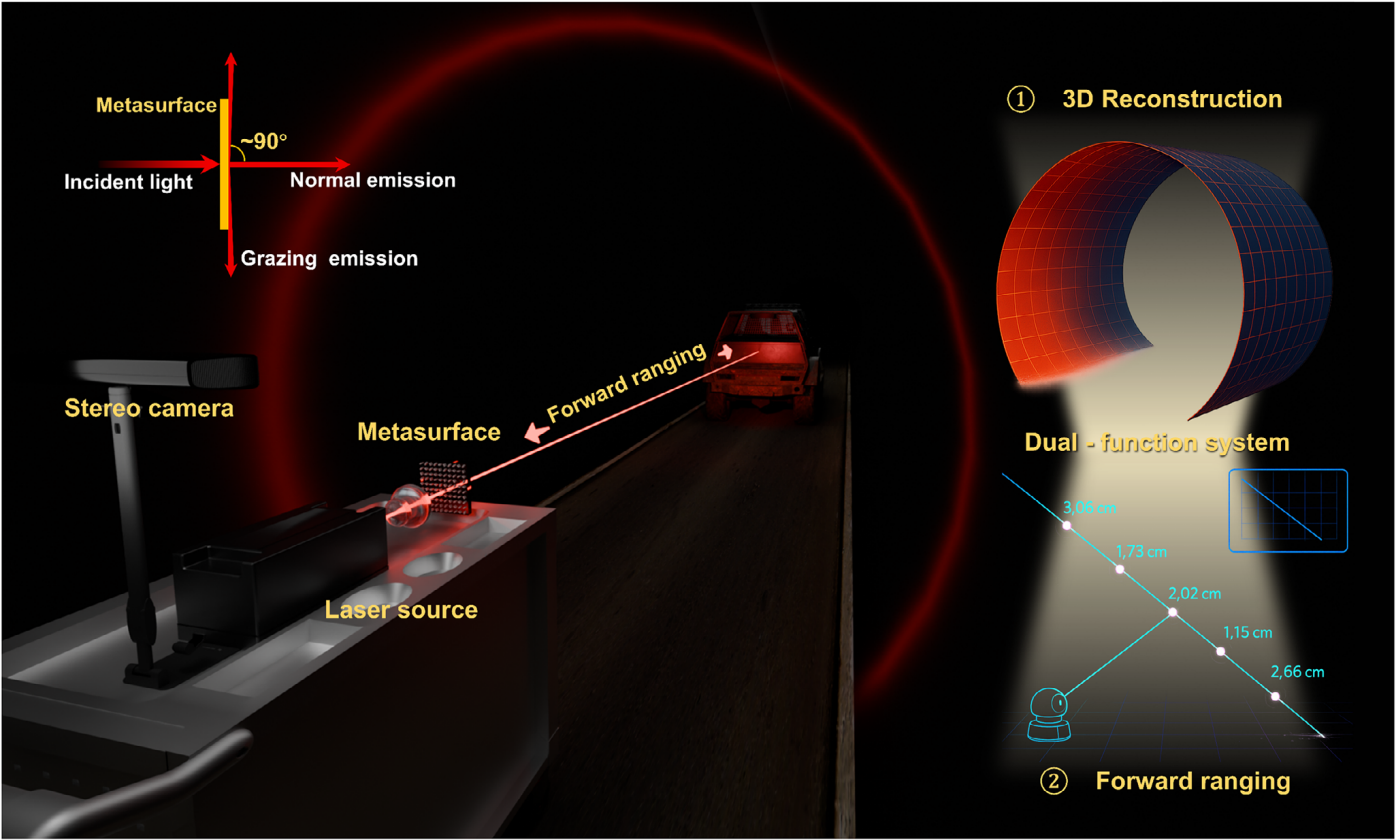
Adaptive structured light based on metasurfaces for multi‐scene 3D reconstruction and forward ranging. The device employs a metasurface‐based structured light system, where adaptive structured light is generated through grazing emission, and a central bright spot is formed through normal emission. These patterns are captured by a camera to compute 3D scene information and forward distance data. The system can be fully integrated onto a mobile platform, enabling multifunctional measurements across multiple scenarios with a single device.

In this paper, we present a solution that integrates metasurfaces with a multimodal point cloud enhancement reconstruction algorithm to develop a metasurface‐based adaptive structured light system, achieving multi‐scene 3D reconstruction and forward ranging with 30 fps. For structured light projection, the system is composed of a single metasurface and utilizes grazing and normal emission light to generate adaptive closed‐line structured light with a central bright spot in the 2π transmission space, without requiring any MEMS or prisms. On the information reception side, the system employs a binocular camera to simultaneously and in real time acquire light spot data for both 3D reconstruction and forward ranging, eliminating the necessity for dedicated distance measurement units. For algorithm design, the multimodal point cloud enhancement reconstruction algorithm integrates the multimodal 3D reconstruction method with the Axis‐Guided Cloud Augmentation algorithm to guarantee robustness in complex environments. The multimodality comprises image modalities (stereo grayscale images and structured light patterns), motion modalities (six‐axis Inertial Measurement Unit (IMU) data), and spatial‐geometric modalities (3D point clouds). In the post‐processing phase, the global motion direction provided by the IMU is utilized to slice and reorder the point cloud, introducing spatial and directional constraints to achieve enhanced reconstruction. The algorithm efficiently addresses model deficiencies caused by environmental factors, greatly enhancing the system's adaptability to changing environmental conditions. To evaluate the system's suitability for multiple scenes, experiments were performed on small rectangular and circular water conveyance pipelines and large‐scale transportation tunnels. As shown in **Figure** **1**, the laser beam passing through the metasurface produces adaptive closed‐line structured light with a central bright spot that matches the tunnel cross‐sectional shape. Using a binocular camera to capture light spot data and transmit it to a computing device, the system achieves real‐time acquisition of 3D information on tunnel walls and distance data near the central bright spot at 30 fps, integrating 3D reconstruction and forward ranging measurement capabilities.

## 2 Results

### 2.1 Design of the Adaptive Closed‐Line Structured Light Metasurface with a Central Bright Spot

**Figure 2:**
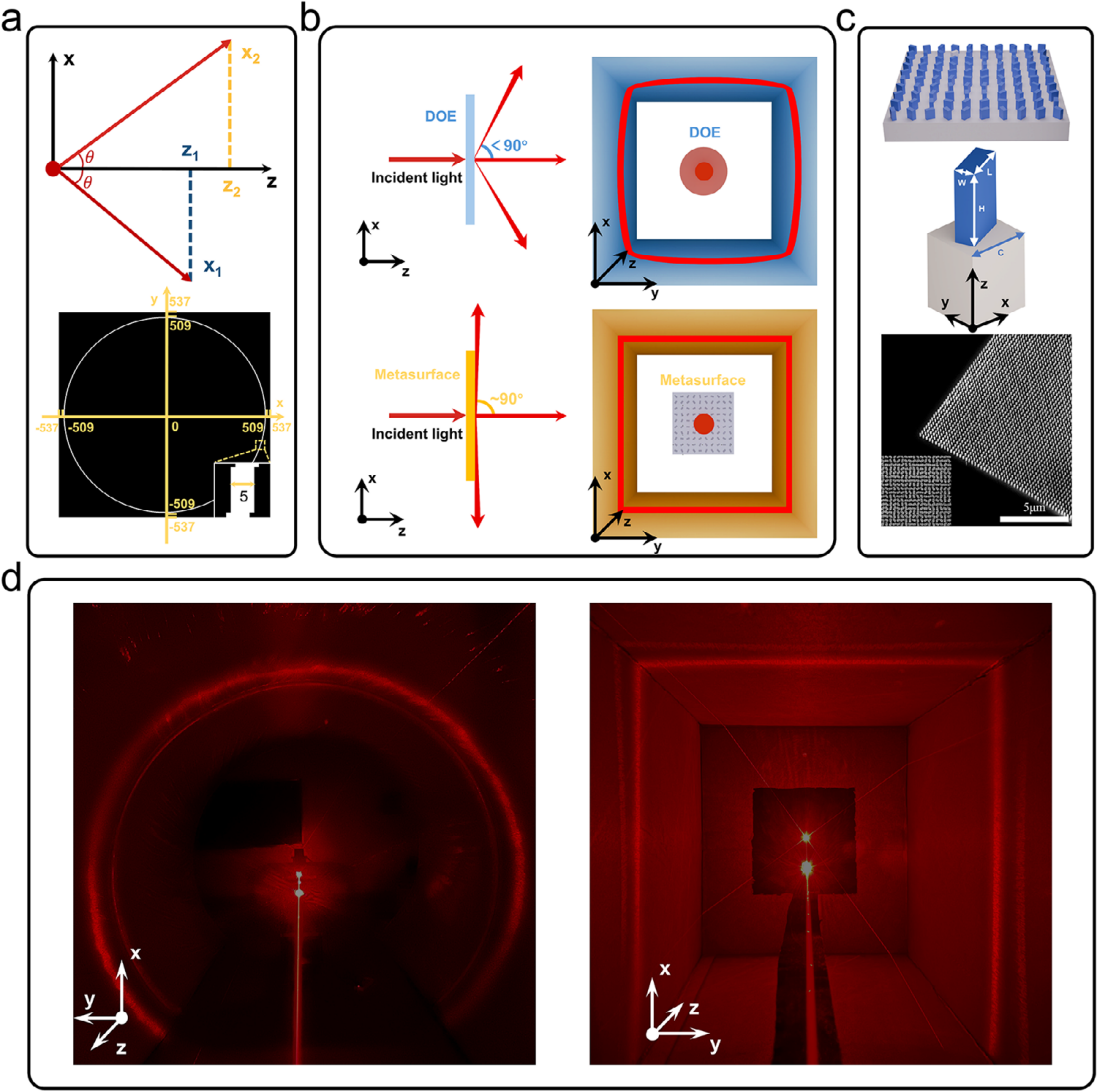
Metasurface design, fabrication, and structured light projection. a) The upper image is a diagram of the diffraction angle design principle. The lower image is the target image, with a local magnified view in the lower right corner. b) Performance comparison of DOE and metasurface, with which one can see obvious geometric distortion of DOE and no distortion of metasurface, thanks to the grazing light emission generated by the metasurface. c) The upper image is a top‐down schematic of the metasurface. The middle image is a diagram of the metasurface unit structure. The lower image is a SEM image of the fabricated metasurface sample, with a detailed magnification displayed in the lower left corner. d) Real‐world images of adaptive structured light in different scenarios. (circular and rectangular cross‐sections, viewpoints in opposite directions along the z‐axis reveal the directional response of the structured light).

To build a 3D reconstruction system adaptable to different tunnel cross‐sectional shapes, it is essential to ensure that the structured light adapts to the tunnel cross‐sectional shape. Specifically, the horizontal position *z* of the light spot must remain consistent at different heights *x*. The deflection angle 𝜃 is the critical parameter in this optical design. The upper part of **Figure** **2**a illustrates the relationship between the 𝜃 and two parameter sets, the vertical distance *x*_1_ of the light spot to the light source's horizontal plane corresponds to the horizontal distance *z*_1_, while *x*_2_ corresponds to *z*_2_. It can be seen from the figure that they adhere to the following relationship,

(1) $$z_{1}=\frac{x_{1}}{\tan\theta }\;$$

(2) $$z_{2}=\frac{x_{2}}{\tan\theta }\;$$

To ensure consistent horizontal distance *z* under varying heights *x*, the deflection angle 𝜃 needs to approach 90° (grazing emission). This is difficult to achieve with a DOE, making the closed‐line structured light emitted from the DOE prone to geometric distortion, which negatively impacts 3D reconstruction performance, as shown in the square tunnel scenario in the upper part of Figure 2b (see Section S2, Supporting Information). Based on Ref.,^[^ ^25^ ^]^ we can use metasurface gratings to generate grazing emission beams, that is, to generate closed‐line structured light with adaptive capabilities, as shown in the lower part of Figure 2b, to overcome the limitations of DOE. Specifically, for the target pattern design, we aimed to maintain the continuity of the structured light across image frames while accommodate environmental and hardware constraints. Applying the beam diffraction angle equation ($\theta_{m}=arc\sin\left(\frac{m\lambda }{pc}\right)$, where *m* is the diffraction order, *λ* is the wavelength, *p* represents the number of nanobricks per grating period, and *c* denotes the unit spacing), the maximum diffraction order of non‐zero diffraction bright spots was set to 509, and the minimum to 505 in our design. The designed target image is shown in the lower part of Figure 2a, with the outermost white ring corresponding to a diffraction order of 509 and the innermost to 505. The maximum diffraction angle for non‐zero bright spots (θ_±509_) is calculated as 87.52°, while the minimum (θ_±505_) is 82.39° (The beam divergence angles of each diffraction order is provided in Section S3, Supporting Information). Although it falls short of the theoretical ideal of 90°, by considering practical constraints such as imaging distance and camera field of view, an adaptive closed‐line structured light system can still be constructed to meet practical needs (see Section S4, Supporting Information). Notably, the zero‐order diffraction light from the normal emission will form a central bright spot along the axis of the adaptive closed‐line structured light. Consequently, an adaptive closed‐line structured light with a central bright spot can be designed using this theory to fulfill the dual requirements of 3D reconstruction and forward ranging.

To achieve this design goal, a high‐transmission geometric phase metasurface composed of subwavelength brick unit structures was designed, as shown in Figure 2c. In this study, the metasurface was designed for an operating wavelength of *λ* = 633 nm, employing amorphous silicon with a high refractive index (*n* = 3.227) and a low extinction coefficient (κ = 0.04) to construct the brick unit structures. The substrate material of the metasurface is silica. A schematic of the nanobrick structure is shown in Figure 2c, with parameters as follows: length *L* = 190  nm, width *W* = 110  nm, height *H* = 355  nm, and unit spacing *C* = 300  nm.

The nanobrick unit structure with these parameters achieves a circularly polarized light transmission efficiency of up to 64% (see Section S5, Supporting Information), indicating that the metasurface constructed with this nanobrick unit structure can achieve high transmittance. (The experimentally measured diffraction efficiency is provided in Section S6, Supporting Information) Geometric phase metasurfaces enable double‐angle phase modulation of light waves by adjusting the orientation angles of the unit structures.^[^ ^25^ ^]^ The required phase distribution was computed using the Gerchberg‐Saxton algorithm. A metasurface sample containing 1075×1075 unit structures was fabricated using standard electron beam lithography, and its scanning electron microscope image is shown in Figure 2c. Figures 2d show the diffraction patterns formed on circular and rectangular cross‐sections when the fabricated metasurface is illuminated by incident light with a wavelength of 633 nm. The results demonstrate that the closed‐line structured light from grazing emission via the metasurface can closely fits the contours of both circular and rectangular cross‐sections, effectively achieving adaptation to different tunnel morphologies. The absence of light at the bottom is due to the occlusion by the metasurface clamping tool (see Section S7, Supporting Information).

### 2.2 Theoretical Principles of Forward Ranging and Scene 3D Information Calculation

**Figure 3:**
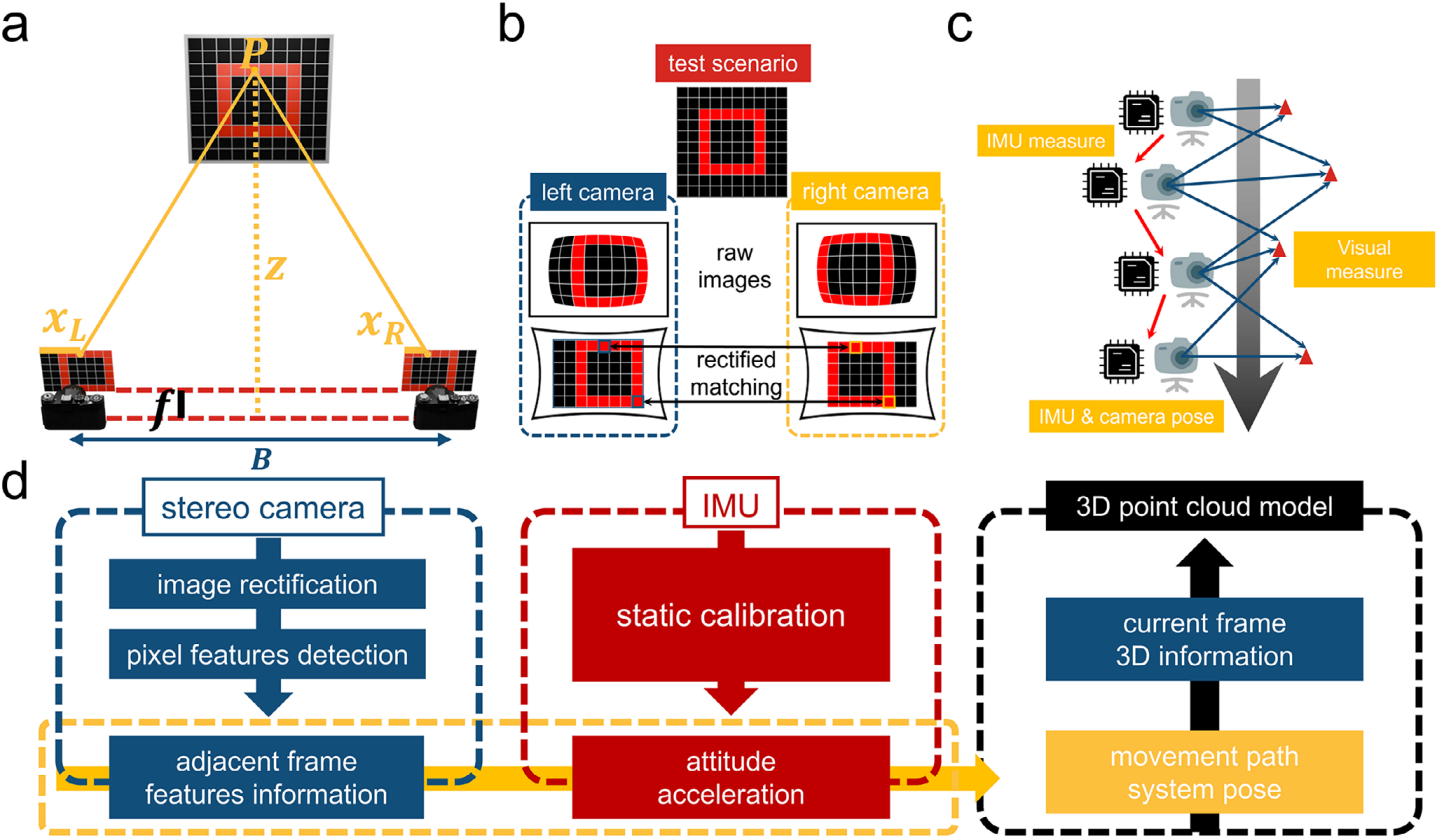
3D information calculation principles and system framework. a) Diagram of binocular camera ranging. b) Camera image correction and alignment of matching pixels to the same horizontal line. c) Combined IMU and camera vision calculation of camera pose and system trajectory, with red arrows showing system motion trajectory and red triangles marking feature pixels. d) Multimodal 3D reconstruction algorithm flow.

To fully utilize the structured light from grazing emission via the metasurface and mitigate the effects of wide‐angle distortion and motion on measurements, a binocular system comprising two global shutter sensors and 150° field‐of‐view wide‐angle lenses was implemented for 3D reconstruction and forward ranging. As shown in **Figure** **3**a, the left and right cameras capture the scene separately, resulting in two slightly shifted images due to the difference in viewpoints. A point P in the scene projects onto the left and right images, with the disparity *d* defined as *d* = *x*_L_ − *x*_R_, representing the horizontal pixel offset between the projections of the same 3D point in the left and right camera images, where *x*_L_ and *x*_R_ represent the horizontal coordinates of the projection points in the left and right images. Through geometric analysis (see Section S8, Supporting Information), the disparity is transformed into distance information between point *P* and the imaging plane. With a baseline length *B* between the binocular camera and a focal length *f*, the distance *z* to the spot from the binocular camera system is calculated as,

(3) $$z=\frac{f\cdot B}{d}$$

This principle serves as both a critical method for forward ranging and the basis for calculating scene depth in 3D reconstruction. This method enables the real‐time extraction of scene depth information from the image disparities between the left and right views, offering technical support for developing an integrated 3D reconstruction and forward ranging system.

In practical scenes, the massive amount of pixel data makes direct global searches for the corresponding pixel of point *P* inefficient. Thus, a pixel row alignment method is utilized to reduce the computation range and enhance efficiency (see Section S9, Supporting Information). As depicted in Figure 3b, geometric modeling and correction were applied to the images using the camera intrinsic parameters (see Section S10, Supporting Information), confining the computation range to a single horizontal line to enhance matching efficiency. Once the matching range is defined, the Semi‐Global Matching algorithm is employed as a similarity metric to improve matching accuracy and robustness to illumination variations (see Section S11, Supporting Information). During forward ranging, the corresponding pixels of point *P* are matched in the left and right images, and the disparity value is obtained. Using binocular ranging geometry, along with known camera intrinsic parameters and baseline length, the distance of the point in 3D space is calculated using Equation (3). For 3D reconstruction, precise camera pose estimation is necessary to construct a 3D spatial map. The geometry between adjacent frames is determined via feature point matching, and relative pose estimation is performed using the essential matrix. Leveraging the binocular camera intrinsic parameter matrix, the singular value decomposition of the essential matrix yields the rotation matrix and translation vector, enabling the computation of camera pose changes. This process is vital for achieving accurate dynamic reconstruction, as only precise pose estimation ensures the correct integration of temporal data to construct a consistent 3D scene. To precisely compute changes in acceleration and angular velocity during device motion, we introduced an Inertial Measurement Unit (IMU) module, as shown in Figure 3c. The IMU monitors real‐time acceleration and angular velocity, combining this data with visual sensors to aid pose estimation through visual‐inertial fusion. In situations where visual features are unstable, IMU‐derived prior information supports accurate pose estimation, greatly improving system robustness and ensuring reliable pose data for 3D reconstruction. We developed a computational framework integrating the aforementioned algorithms. Hardware‐based timing synchronization and precise calibration ensure that the binocular camera provides consistently paired left and right images to the algorithm. Corrected binocular camera images serve for pixel feature extraction, with motion information derived by analyzing feature changes between consecutive frames, illustrated in Figure 3d. Through static calibration and denoising, the IMU supplies angular velocity and acceleration data for the system (see Section S12, Supporting Information). Finally, the system integrates binocular camera and IMU data to compute the motion trajectory and pose (see Sections S13 and S14, Supporting Information). Additionally, the 3D point cloud from the current frame is fused with the system's trajectory to incrementally construct a complete 3D point cloud model. In the process of 3D reconstruction and forward ranging, the metasurface‐based integrated system is engineered to align its motion path within the tunnel with the scene's longitudinal axis. Based on this, by using wide‐angle lenses and the designed computational framework, the system fully captures the light spot data of adaptive closed‐line structured light with a central bright spot in a single frame, enabling integrated 3D reconstruction and forward ranging within the tunnel space.

### 2.3 3D Reconstruction for Rectangular and Circular Pipeline Environments

**Figure 4:**
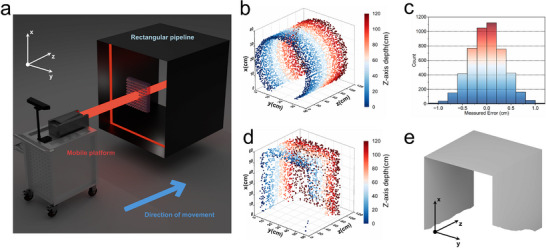
Experiments on 3D modeling of circular and rectangular cross‐section scenes. a) The experimental scene. b) 3D point cloud representation of the circular pipeline. c) Radius measurement error value distribution in the 3D point cloud. d) 3D point cloud representation of the rectangular pipeline. e) 3D point cloud surface reconstruction of the rectangular pipeline.

To verify the system's suitability for various scenes, experiments were performed on circular and rectangular pipeline environments. The circular pipeline has a diameter of 400 mm, while the rectangular pipeline features a side length of 600 mm. As shown in **Figure** **4**a, the metasurface was positioned at the geometric center of the pipeline's cross‐section, with the binocular camera located behind it. The hardware setup was mounted on a cart moving along the pipeline axis. Figures 2d show the light spot distribution of the adaptive structured light in circular and rectangular scenes, respectively. The multimodal 3D reconstruction algorithm calculated the internal 3D information of the pipe in real time at a speed of 30 fps. The Video S1 (Supporting Information) demonstrates the real‐time computation of internal 3D information for the circular pipeline depicted in Figure 4b. Figure 4b,d displays the 3D point cloud models generated by the system in different scenarios. It can be observed that the point cloud models accurately reconstructed the morphological features of the pipes. In the circular pipeline's 3D point cloud, the system successfully captured the circumferential inner wall structure, while in the rectangular pipeline, the model highlighted well‐defined edges and planes, showcasing the system's adaptability to various scenes. An error analysis was performed on the circular pipeline's point cloud data to quantify the system's depth calculation accuracy. The measured radius error values of each point in the point cloud (shown in Figure 4c) were compared with the actual radius of 200 mm, yielding a Root Mean Square Error (RMSE) of 3.5 mm. The measurement error mainly arises from the light spot and camera jitter during motion. To verify the system's accuracy in spatial position calculations, plane fitting analysis was conducted on the 3D point cloud data of the rectangular pipe. Figure 4e depicts the results of the plane fitting analysis. The plane fitting analysis revealed that the point cloud consists of three orthogonal planes. These results confirm the system's reliability in calculating point cloud spatial positions and its ability to preserve geometric relationships between scene regions through adaptive structured light, which is essential for complex 3D reconstruction tasks. The results from both circular and rectangular experimental scenarios indicate that the system has excellent adaptability across multiple scenarios.

### 2.4 Integrated 3D Reconstruction and Forward Ranging in Complex Environments

**Figure 5:**
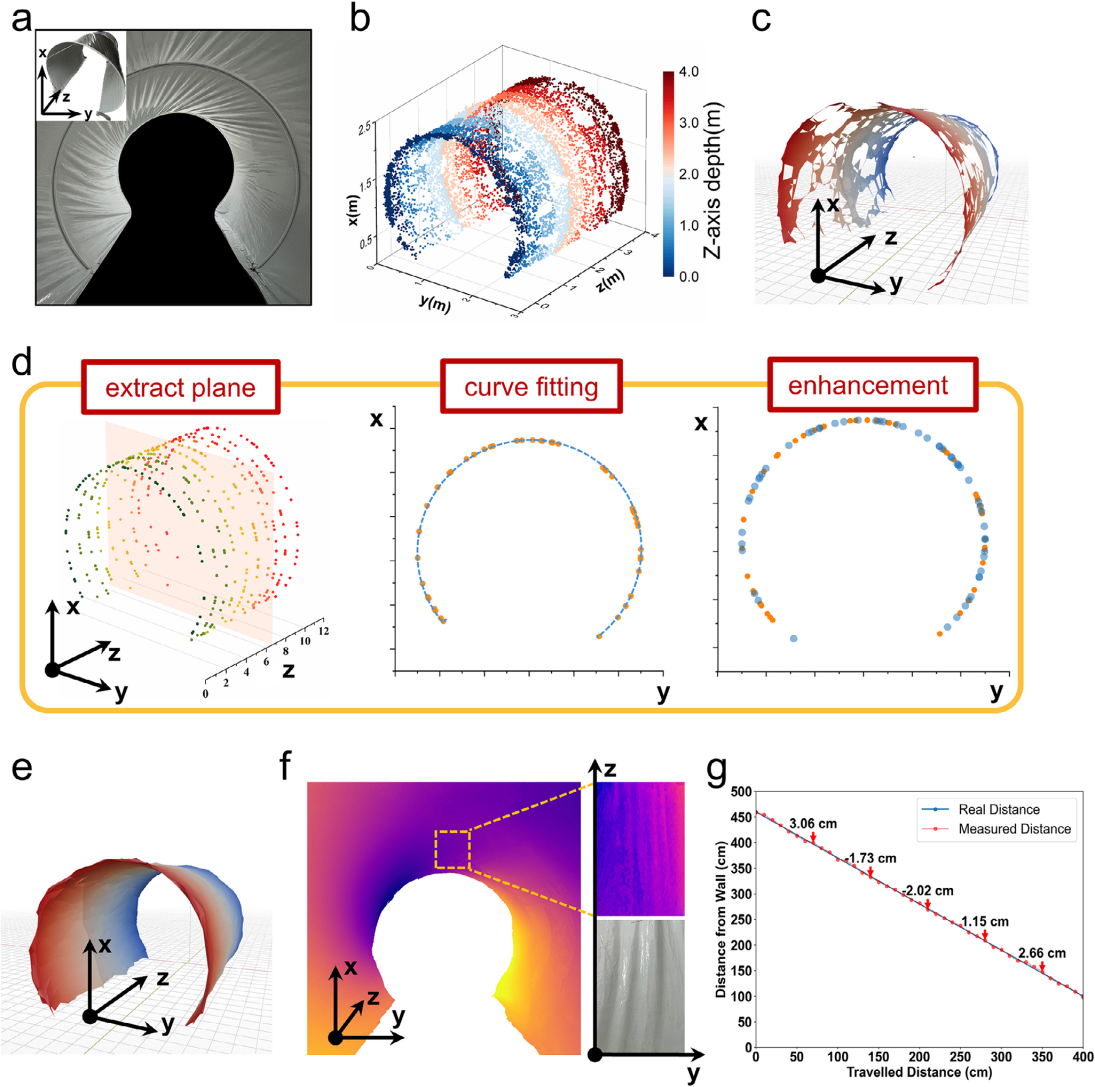
Tunnel scene measurements and 3D model quality improvement with point cloud enhancement algorithms. a) Overall and internal view of the tunnel scene. b) Tunnel 3D point cloud model. c) Surface reconstruction model before optimization with enhancement algorithms. d) Axial‐guided point cloud enhancement algorithm workflow. e) Surface reconstruction model optimized with the enhancement algorithm. f) Depth distribution inside the tunnel, with color gradients indicating distance changes from the central axis. The small figure in the upper right is a local magnification of the depth distribution, and the lower right is a picture of the actual wrinkles. g) Measuring cart‐to‐wall distances using the central bright spot from zero‐order diffraction, with numbers above red arrows showing discrepancies between measured and actual values.

To verify the system's adaptability in a large‐scale, uneven and scene with reflective tunnel walls, we tested 3D reconstruction and forward ranging in a tunnel with a diameter of 3 m, height of 2.4 m, and length of 4 m, as illustrated in **Figure** **5**a. In the experiment, a laser was used to illuminate the metasurface located at the center of the tunnel cross‐section, generating adaptive closed‐line structured light with a central bright spot in the 2π transmission space, as shown in the conceptual diagram in Figure 1. The 3D point cloud model shown in Figure 5b indicates that the system successfully reconstructed the tunnel's overall contour and prominent features (The measurement time is detailed in Supporting Information S15). However, some voids were observed in the model. These voids were mainly caused by the reflective properties of the tunnel construction materials and the shadowing effect of some uneven structures. As a result, the voids in the point cloud can impact both the fidelity and spatial continuity of the subsequent reconstructed model (as illustrated in Figure 5c).

To enhance model quality, the unique capabilities of metasurface adaptive structured light were utilized to develop an axial‐guided point cloud enhancement algorithm. The algorithm partitions the 3D point cloud into axial planes and incrementally fills gaps within each plane for global optimization. As shown in Figure 5d, the algorithm first partitions the point cloud data along the z‐axis. Within each partitioned plane, it applies a distance minimization and direction selection method to construct an ordered point set. Specifically, the current reference point *P*_cur_ and the remaining points *P*_rem_ not yet in the ordered set are considered. The next point following *P*_cur_ should be close enough to ensure spatial adjacency in the set. A distance threshold *d* is defined to select candidate points *P*_i_, and points meeting Equation (4) are considered candidates.

(4) $$\left\|P_{i}-P_{cur}\right\|<d,P_{i}\in P_{rem}$$

Following distance filtering, the next point is selected from candidates as the one most aligned with the previous direction vector to ensure directional consistency. The direction vector *V* between the *P*_cur_ and the previous point *P*_pre_ is *V* = *P*_cur_ − *P*_pre_, while for *P*_i_, it is *P*_i_ − *P*_cur_. Their directional consistency is calculated as follows,

(5) $$\cos\theta =\frac{V\cdot \left(P_{i}-P_{cur}\right)}{∥V∥\cdot ∥P_{i}-P_{cur}∥}$$

The closer the value of cos*θ* is to 1, the better the alignment of the vectors. The candidate point with the highest cos*θ* is selected as *P*_next_, ensuring directional consistency. In areas with sparse point clouds, spline interpolation is applied to enhance density before proceeding with further processing. Using the ordered point set, curves derived from point set fitting are employed to fill the missing point clouds, resulting in an enhanced point cloud model. It is important to note that segmenting the 3D point cloud data from distorted structured light could result in non‐coplanar points being wrongly mapped to the same plane, affecting the accuracy of cross‐sectional fitting. Following optimization by the point cloud enhancement algorithm, the resulting 3D tunnel model (Figure 5e) fills the gaps present in the unoptimized model (Figure 5c) and exhibits a more continuous surface with visible fold features (Figure 5f). These enhancements greatly improve the fidelity and surface continuity of the reconstructed model, aligning it more closely with the actual tunnel and offering robust support for 3D reconstruction in complex scenes. In addition to the 3D reconstruction experiments using the structured light from grazing emission, we also conducted distance measurement experiments using the zeroth‐order light from normal emission. Figure 5g illustrates the experimental forward ranging results on the tunnel exit wall during motion. In the forward ranging experiment, the camera was mounted 4.6 meters from the tunnel exit wall to capture light data surrounding the central bright spot for forward ranging computation. The system conducted measurements every 10 cm during the experiment, collecting a total of 40 data sets with an RMSE of 0.022 m. The experiments fully validated the system's integrated 3D reconstruction and forward ranging capabilities in complex scenes, confirming its feasibility and effectiveness in dynamic environments. The system's integrated design allows seamless collaboration between the IMU, vision sensors, 3D reconstruction, and forward ranging technologies, boosting processing efficiency, minimizing hardware and algorithm redundancies, and improving real‐time performance and robustness.

## 3 Discussion

We present a novel 3D reconstruction and forward ranging system integrating metasurfaces with a multimodal point cloud enhancement algorithm. The system enables both grazing and normal emission of light by performing high‐precision phase modulation of the incident light using a single metasurface, thereby generating an adaptive closed‐line structured light with a central bright spot in the 2π transmission space. This method eliminates the tendency of the DOE to cause spot distortion while simultaneously generating spot information suitable for forward ranging. Through a multimodal 3D reconstruction algorithm, the system performs real‐time 3D scene calculations at 30 fps and employs the metasurface‐generated central bright spot for forward ranging. In reflective or irregular environments, the axial‐guided point cloud enhancement algorithm ensures high‐quality 3D reconstruction.

Compared with existing representative 3D metasurface imaging approaches, the proposed method offers enhanced adaptability in structured light emission angle control and system integration (see Supporting Information S16). For instance, Luo et al. introduced a monocular structured light system for wide field‐of‐view projection,^[^ ^35^ ^]^ Liu et al. leveraged binocular metalenses with neural networks to enhance edge modeling,^[^ ^36^ ^]^ and Xu et al. proposed a fully optical identification structure.^[^ ^37^ ^]^ However, most of these methods focus on normal regions or static targets, making it difficult to achieve both sidewall coverage and real‐time performance in tunnel‐like environments. Compared with LiDAR, photogrammetry, and traditional structured light systems, this system demonstrates significant advantages in real‐time performance and adaptability to various scenarios. In the three tested scenes, the system captures the entire cross‐sectional light spot within a single frame, eliminating the need for block scanning and greatly improving real‐time depth data acquisition and computational efficiency. The compactness and integrability of metasurfaces further enable deployment on diverse mobile platforms, making them ideal for dynamic measurement tasks in complex environments. Additionally, in reflective conditions, the synergy between adaptive structured light and the axial‐guided point cloud enhancement algorithm overcomes the environmental dependencies of traditional approaches. Most importantly, the system accomplishes both 3D reconstruction and forward ranging using a single hardware platform, significantly enhancing integration and simplifying maintenance. This system is also extendable to the infrared spectrum, leveraging its efficiency in low‐light conditions, high penetration capability, and interference resistance to bolster adaptability and usability in complex scenes. These innovations not only overcome the limitations of traditional methods and greatly simplify the system design but also pave the technological path for multifunctional applications of the same system under multiple scene conditions.

The findings of this study offer a viable approach for next‐generation ultra‐compact 3D reconstruction platforms and reveal the extensive applicability of metasurface technology in light field control and sensing. In the future, this system holds significant promise for advancing fields such as construction engineering and smart transportation by incorporating smart materials and deep learning algorithms, opening new interdisciplinary avenues for innovation in related domains.

## 4 Experimental Section

### Experimental Setup

The hardware system includes a laser (MDL‐III‐633L), 1.5× beam expander, an IMU module (BMI270), and a binocular camera made up of two OV9282 sensors with 150° lenses. The beam expander ensures complete coverage of the metasurface by the laser beam. The binocular camera and IMU module were integrated onto a printed circuit board, connected to a computer via USB 3.0, and positioned behind the metasurface to record the central bright spot and structured light images in the transmission space.

Considering computational resource constraints and the need for stable synchronization between the left and right images, the camera frame rate was set to 30 fps, with the resolution adjusted to 960×720. This resolution setting helps crop out redundant image margins and exclude irrelevant information outside the target scene, thereby reducing the computational burden for image processing and 3D reconstruction while improving overall system efficiency (see Supporting Information S17).

The primary bottleneck in system frame rate originates from the computational load of image processing and point cloud generation, rather than from limitations in the camera, metasurface, or data transmission hardware. All processing tasks—including image acquisition, distortion correction, reconstruction, and point cloud rendering—are executed in a multi‐threaded CPU environment (AMD Ryzen 5 5600X), without GPU acceleration. Each frame takes ≈30–35 milliseconds to process. Increasing the frame rate would lead to data accumulation in the processing queue, potentially causing increased system latency or frame drops.

### Calibration of Camera and IMU

The calibration process was conducted using the Kalibr tool.^[^ ^38^, ^39^ ^]^ The process begins with binocular camera calibration, which involves determining the camera intrinsic parameters, extrinsic parameters, and distortion coefficients. The camera relies on these parameters to project 3D points from the real world onto 2D image points. Camera calibration was performed using an AprilTag‐based grid calibration board (AprilGrid). The AprilGrid was shown on a matte computer screen, and images were taken from four different distances (50, 70, 90, and 100 cm) at various angles for calibration. This calibration yielded the intrinsic parameters and relative poses of the left and right cameras. Subsequently, IMU calibration was performed, recording accelerometer and gyroscope data while keeping the camera stationary for more than two hours. Static data were utilized to estimate the accelerometer's bias and scale factor and to evaluate its noise characteristics and deviations. Finally, binocular camera and IMU joint calibration were performed to acquire the extrinsic parameters relating the two systems.

## Conflict of interest

The authors declare no conflict of interest.

## Supporting information



Supporting Information

Supplemental Video 1

## Data Availability

The data that support the findings of this study are available from the corresponding author upon reasonable request.

## References

[1] T. T. Nguyen , D. C. Slaughter , N. Max , J. N. Maloof , N. Sinha , Sensors 2015, 15, 18587. 26230701 10.3390/s150818587 PMC4570338

[2] C. Albitar , P. Graebling , C. Doignon , in IEEE 11th Int. Conf. Comput. Vision , IEEE, Rio de Janeiro, Brazil 2007, pp. 1–6.

[3] B. Cui , W. Tao , H. Zhao , Remote Sens. 2021, 13, 4457.

[4] S.‐P. Yang , Y.‐H. Seo , J.‐B. Kim , H. Kim , K.‐H. Jeong , Micro Nano Syst. Lett. 2019, 7, 8.

[5] X. Zhang , S. J. Koppal , R. Zhang , L. Zhou , E. Butler , H. Xie , Opt. Express 2016, 24, 3479. 26907006 10.1364/OE.24.003479

[6] N. Ullah , R. Zhao , L. Huang , Micromachines 2022, 13, 1025. 35888842 10.3390/mi13071025 PMC9322754

[7] J. Qin , S. Jiang , Z. Wang , X. Cheng , B. Li , Y. Shi , D. P. Tsai , A. Q. Liu , W. Huang , W. Zhu , ACS Nano 2022, 16, 11598. 35960685 10.1021/acsnano.2c03310

[8] S. Sun , Q. He , J. Hao , S. Xiao , L. Zhou , Adv. Opt. Photonics 2019, 11, 380.

[9] S. Wang , S. Wen , Z.‐L. Deng , X. Li , Y. Yang , Phys. Rev. Lett. 2023, 130, 123801. 37027878 10.1103/PhysRevLett.130.123801

[10] Z. Feng , T. Shi , G. Geng , J. Li , Z.‐L. Deng , Y. Kivshar , X. Li , eLight 2023, 3, 21.

[11] Q. Chen , G. Qu , J. Yin , Y. Wang , Z. Ji , W. Yang , Y. Wang , Z. Yin , Q. Song , Y. Kivshar , S. Xiao , Nat. Nanotechnol. 2024, 19, 1000. 38561429 10.1038/s41565-024-01636-y

[12] C. Liang , Q. Dai , T. Huang , H.‐C. Liu , Z. Guan , R. Fu , J. Tao , Z. Li , S. Yu , G. Zheng , J. Materiomics 2024, 10, 811.

[13] M. A. Ansari , H. Ahmed , Y. Li , G. Wang , J. E. Callaghan , R. Wang , J. Downing , X. Chen , Light:Sci. Appl. 2024, 13, 224. 39223113 10.1038/s41377-024-01565-4 PMC11369200

[14] T. Badloe , J. Kim , I. Kim , W.‐S. Kim , W. S. Kim , Y.‐K. Kim , J. Rho , Light:Sci. Appl. 2022, 11, 118.35487908

[15] G. Zheng , H. Mühlenbernd , M. Kenney , G. Li , T. Zentgraf , S. Zhang , Nat. Nanotechnol. 2015, 10, 308. 25705870 10.1038/nnano.2015.2

[16] K. Pan , X. Wu , P. Li , S. Liu , B. Wei , D. Li , D. Yang , X. Chen , J. Zhao , D. Wen , Nano Lett. 2024, 24, 6761. 38775803 10.1021/acs.nanolett.4c01490

[17] K. Pan , X. Wu , L. Zhou , B. Wei , D. Li , S. Liu , P. Li , D. Yang , J. Zhao , D. Wen , Opt. Lett. 2023, 48, 4217. 37581996 10.1364/OL.499012

[18] Z. Li , I. Kim , L. Zhang , M. Q. Mehmood , M. S. Anwar , M. Saleem , D. Lee , K. T. Nam , S. Zhang , B. Luk'yanchuk , Y. Wang , G. Zheng , J. Rho , C.‐W. Qiu , ACS Nano 2017, 11, 9382. 28898048 10.1021/acsnano.7b04868

[19] N. A. Rubin , G. D'Aversa , P. Chevalier , Z. Shi , W. T. Chen , F. Capasso , Science 2019, 365, aax1839. 10.1126/science.aax1839 31273096

[20] Y. Li , M. A. Ansari , H. Ahmed , R. Wang , G. Wang , X. Chen , Sci. Adv. 2023, 9, adj6675. 10.1126/sciadv.adj6675 PMC10664995 37992179

[21] X. Jing , Y. Li , J. Li , Y. Wang , L. Huang , Nanophotonics 2023, 12, 1923. 39635136 10.1515/nanoph-2023-0112 PMC11501738

[22] D. B. Conkey , R. P. Trivedi , S. R. P. Pavani , I. I. Smalyukh , R. Piestun , Opt. Express 2011, 19, 3835. 21369208 10.1364/OE.19.003835

[23] Z. Zhang , Q. Sun , A. Qu , M. Yang , Z. Li , Opt. Lett. 2024, 49, 6325. 39485478 10.1364/OL.538443

[24] Z. Zhang , A. Qu , M. Yang , Z. Li , T. Huang , S. Chen , H. Cheng , S. Yu , G. Zheng , Laser Photonics Rev. 2025, 19, 2401120.

[25] Z. Li , Q. Dai , M. Q. Mehmood , G. Hu , B. L. Yanchuk , J. Tao , C. Hao , I. Kim , H. Jeong , G. Zheng , S. Yu , A. Alù , J. Rho , C.‐W. Qiu , Light:Sci. Appl. 2018, 7, 63. 30245810 10.1038/s41377-018-0064-3 PMC6134062

[26] S. Fekete , M. Diederichs , M. Lato , Tunnelling Underground Space Technol. 2010, 25, 614.

[27] B. V. Farahani , F. Barros , P. J. Sousa , P. P. Cacciari , P. J. Tavares , M. M. Futai , P. Moreira , Tunnelling Underground Space Technol. 2019, 91, 102995.

[28] P. An , K. Fang , Q. Jiang , H. Zhang , Y. Zhang , Sensors 2021, 21, 922. 33573128 10.3390/s21030922 PMC7866559

[29] M. Scaioni , L. Barazzetti , A. Giussani , M. Previtali , F. Roncoroni , M. I. Alba , Earth Sci. Inf. 2014, 7, 83.

[30] M. Schaffer , M. Grosse , B. Harendt , R. Kowarschik , Opt. Lett. 2011, 36, 3097. 21847172 10.1364/OL.36.003097

[31] P. Zhou , J. Zhu , W. Xiong , J. Zhang , Appl. Opt. 2021, 60, 5925. 34263814 10.1364/AO.430101

[32] Z. Cai , G. Pedrini , W. Osten , X. Liu , X. Peng , Opt. Lett. 2020, 45, 3256. 32538956 10.1364/OL.393911

[33] Y. Feng , P. Li , G. Tao , R. Wu , J. Lin , X. Liu , L. Chen , Meas. Sci. Technol. 2024, 36, 015428.

[34] S. Lv , Q. Sun , Y. Zhang , Y. Jiang , J. Yang , J. Liu , J. Wang , Opt. Lett. 2020, 45, 204.

[35] Y. Luo , X. Li , R. Zhang , Y. Guo , M. Pu , Y. Fan , Q. Zhang , Q. He , J. Che , Z. Zhao , X. Luo , ACS Appl. Mater. Interfaces 2024, 16, 39906. 39024478 10.1021/acsami.4c09254

[36] X. Liu , J. Zhang , B. Leng , Y. Zhou , J. Cheng , T. Yamaguchi , T. Tanaka , M. K. Chen , Opto‐Electron. Sci. 2024, 3, 230033.

[37] D. Xu , W. Xu , Q. Yang , W. Zhang , S. Wen , H. Luo , Opto‐Electron. Sci. 2023, 6, 230120.

[38] J. Rehder , R. Siegwart , P. Furgale , IEEE Trans. Rob. 2016, 32, 383.

[39] Q. Zhang , R. Pless , in IEEE/RSJ Int. Conf. Intell. Rob. Syst. (IROS) , IEEE, Sendai, Japan 2004, 3, pp. 2301–2306.

