# Supplementary material for: Metasurface‐Driven Adaptive Structured Light: Achieving Integrated Real‐Time 3D Reconstruction and Forward Ranging in Multi‐Scene
Source: Adv Sci (Weinh). 2025 Jul 14;12(39):e07339. doi: 10.1002/advs.202507339 (PMC12533358; doi:10.1002/advs.202507339)
Supplement: Supplementary file 1 — Supporting Information [file ADVS-12-e07339-s001.docx]

Supporting Information

Metasurface-Driven Adaptive Structured Light: Achieving Integrated Real-Time 3D Reconstruction and Forward Ranging in Multi-Scene

1. Comparison with Commercial DOE Components
As shown in Table S1, we list the product model, DOE types, and maximum diffraction angles of commercial DOE products from Holoeye and Highlightoptics (HL). Commercial DOEs generally face limitations in achievable diffraction angles due to constraints in structural periodicity, with only a few products exceeding 50°. In contrast, the metasurface used in this study achieves a maximum diffraction angle of 88°, significantly surpassing the typical upper limits of mainstream commercial DOE designs.A larger diffraction angle not only expands the spatial coverage within a single frame—thus reducing the computational burden of multi-frame stitching—but also provides a robust geometric foundation for light field projection in curved or enclosed environments such as tunnels. Therefore, from the perspectives of field-of-view coverage, system integration, and environmental adaptability, the metasurface demonstrates inherent technical advantages for such applications.

Table S1. Comparison of Maximum Diffraction Angles in Common DOEs

| Model | Type | Maximum Diffraction Angle |
| --- | --- | --- |
| Holoeye-PBS-635-2D5S-11 | 2D dot-array structured light DOE | 28.2° |
| Holoeye-PDZK-645-16-10 | Patterned structured light DOE | 51.4° |
| HL -G3LI-650-67-17 | Multi-line structured light DOE | 33.5° |
| HL -S-905-120×20 | Homogenizing spot DOE | 55° |

2. Comparison between adaptive structured light and conventional laser-generated structured light, and the impact of spot distortion on algorithm performance

Adaptability is one of the core objectives in the design of the structured light system presented in this work. The adaptability refers to the ability to conform to different geometric scenarios. By precisely controlling the diffraction angles and spot distribution of the metasurface, the generated structured light patterns can naturally and geometrically fit various typical tunnel cross-sections. As illustrated in Figure S1a, to ensure that the structured light pattern aligns naturally with the tunnel profile, the design goal is to maintain a consistent horizontal projection distance z at different vertical heights *x*, satisfying 𝑧 = 𝑥 / tan𝜃. When the grazing emission angle 𝜃 approaches 90°, tan𝜃 approaches an infinite value, causing *z* to converge across different x values. This enables geometrically equidistant coverage across the cross-sectional profile and significantly reduces geometric distortions caused by vertical height differences, improving both the coplanarity of the structured light and the consistency of the resulting point cloud. As shown in Figure S1c, we conducted comparative tests in three scenarios with cross-sectional shapes: rectangular, hexagonal, and triangular. The first row displays the results using our proposed adaptive structured light, while the second row shows those generated by a 45° divergence ring-shaped laser. In the yellow boxes, the projected light spots remain straight and undistorted. In contrast, in the green boxes, due to mismatches between the ring-shaped pattern and the underlying geometry (rectangle, hexagon, triangle), along with the relatively small divergence angle, noticeable spot deformation occurs, which adversely affects 3D reconstruction. This is further evidenced by Figure S1b, where the resulting point clouds appear scattered and lose coplanarity, undermining subsequent point cloud enhancement.

The geometric adaptability of the grazing emission structured light substantially reduces spatial distortion, ensuring that matching points extracted from each frame are naturally distributed on a common plane in 3D space. Compared with conventional structured light systems—where projection distortion or warping often causes spatial displacement—this property directly improves the geometric fidelity of the point cloud. In the subsequent point cloud enhancement stage, such inherent coplanarity ensures that the point set in each axial slice naturally satisfies fitting assumptions, eliminating the need to explicitly identify coplanar regions or perform complex pre-grouping. This greatly reduces computational complexity in curve fitting and interpolation. Thus, the grazing emission structured light not only ensures pattern quality at the physical level, but also establishes geometric consistency in the light field, optimizing the organization of point cloud data at the source. Consequently, it significantly reduces the logical complexity and computational overhead of downstream modeling algorithms.


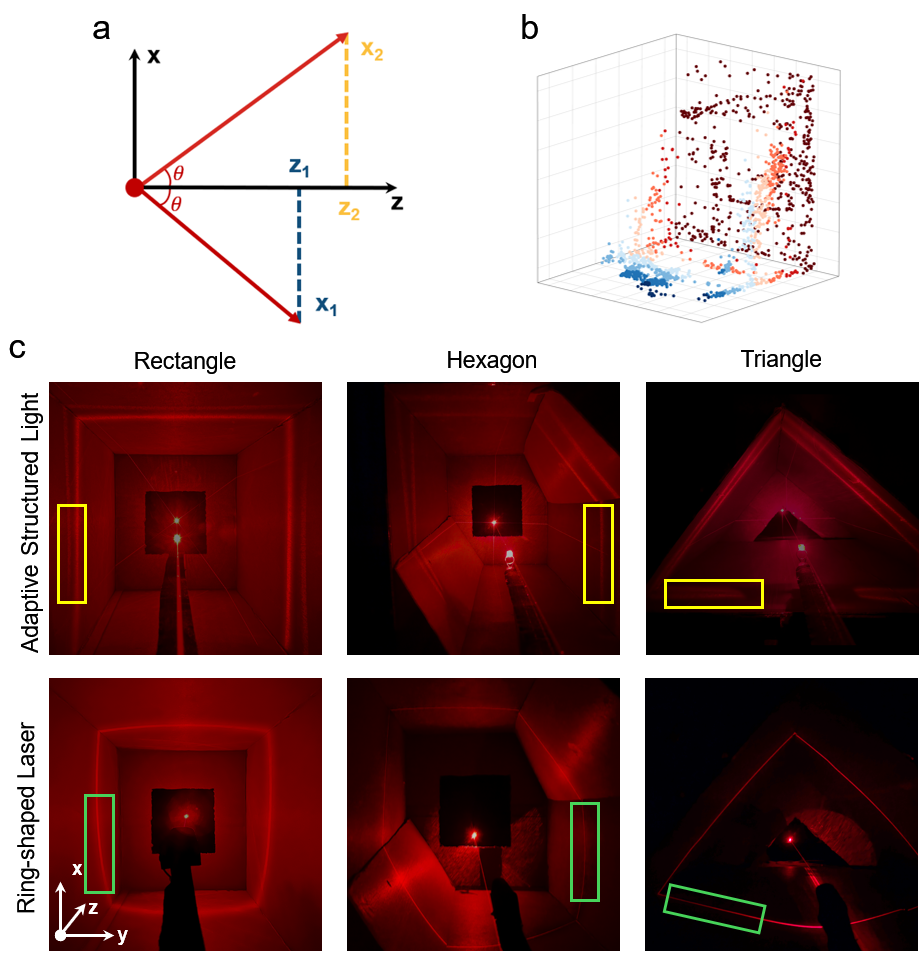


Figure S1. Impact of spot distortion on 3D information computation. a) Schematic diagram of diffraction angle design principle. b) 3D point cloud model of a rectangular box cross-section using a ring-shaped laser projector. c) Comparative tests of adaptive structured light generated by a metasurface and a ring-shaped laser projector under scenes with different cross-sectional shapes.

3. The Impact of Beam Divergence Angle on Reconstruction

The diffraction angles ranging from 82° to 88° mentioned in the text correspond to diffraction orders from the 505th to the 509th. The specific diffraction angles for each order are 82.39°, 83.27°, 84.33°, 85.64°, and 87.52°, respectively. For each diffraction order, the beam divergence angle Δ𝜃 is primarily determined by the size of the grating, and it can be calculated using the formula: Δ𝜃 = 2arcsin(𝜆 / 𝐿), where 𝜆 is the laser wavelength and 𝐿 is the metasurface aperture. In our design, with 𝜆 = 633 nm and 𝐿 = 645 𝜇m, the calculated divergence angle is Δ𝜃 = 0.11°, indicating that the grazing emission beams corresponding to each diffraction order exhibit extremely narrow divergence. Thus, their impact on long-distance 3D reconstruction is negligible. On the other hand, the normal beams mainly originates from incident light that does not undergo polarization conversion—that is, light not modulated by the metasurface phase. Its divergence angle depends on the characteristics of the laser source. The laser used in our system is the MDL-III-633L, which has a specified output divergence of less than 1.0 mrad (approximately 0.057°), which is similarly minimal.In summary, although the grazing emission and normal-emission beams originate from different mechanisms, both exhibit very small divergence angles, and their influence on the practical performance of the system is negligible.

4. Target Pattern Design

Considering the camera parameters and computational device performance, the frame capture rate was set at 30 fps, with a resolution of 960×720. The width of the tunnel model is 3 m, and the frame fits this width perfectly, giving each pixel a physical size of 3 m / 960 = 0.003 m. The tunnel cross-section has a total length of 9.5 m, requiring at least 9.5 m / 0.003m = 3200 bright spots to ensure continuity on the tunnel wall in the camera frame. Therefore, the target ring diameter should be 3200 / π = 1019. According to the diffraction angle formula 𝜃_𝑚_ = arcsin(𝑚𝜆 / 𝑝𝑐), under the condition that the number of bright spots reaches 3200 and the grating period *c* is 300  nm, a diffraction order of 𝑚 = 509 and period *p* = 537 yields a maximum diffraction angle of 88°. Each diffraction order produces a very narrow ring-shaped pattern. Using only a single order would result in a sparse or layered structure, lacking continuity. To address this, we evaluated combinations of multiple adjacent diffraction orders. Experimental results showed that using five consecutive orders from ±505 to ±509 produces a ring width of approximately 10  cm in the physical space, with no obvious gaps or discontinuities. This configuration was identified as the minimal effective combination that balances spatial spot density and visual continuity in the axial direction of the model.

5. Design and Fabrication of the Metasurface

According to the theory of geometric phase metasurfaces, ^[1]^ we use *T*_co_, *T*_cross_, *R*_co_, and *R*_cross_ to represent the efficiencies of transmitted co-polarized circularly polarized(CP), transmitted cross-polarized CP, reflected co-polarized CP, and reflected cross-polarized CP, respectively. The corresponding expressions are,

|  | $\boldsymbol{T}_{\boldsymbol{co}}\mathbf{=}\left\vert\boldsymbol{p}_{\boldsymbol{t}} \right\vert^{\mathbf{2}}\mathbf{=}\left\vert\frac{\boldsymbol{t}_{\boldsymbol{l}}\mathbf{+}\boldsymbol{t}_{\boldsymbol{s}}\boldsymbol{e}^{\boldsymbol{i\delta}_{\boldsymbol{t}}}}{\mathbf{2}} \right\vert^{\mathbf{2}}$ | (S1) |
| --- | --- | --- |
|  | $\boldsymbol{T}_{\boldsymbol{cross}}\mathbf{=}\left\vert\boldsymbol{q}_{\boldsymbol{t}} \right\vert^{\mathbf{2}}\mathbf{=}\left\vert\frac{\boldsymbol{t}_{\boldsymbol{l}}\mathbf{-}\boldsymbol{t}_{\boldsymbol{s}}\boldsymbol{e}^{\boldsymbol{i\delta}_{\boldsymbol{t}}}}{\mathbf{2}} \right\vert^{\mathbf{2}}$ | (S2) |
|  | $\boldsymbol{R}_{\boldsymbol{co}}\mathbf{=}\left\vert\boldsymbol{p}_{\boldsymbol{r}} \right\vert^{\mathbf{2}}\mathbf{=}\left\vert\frac{\boldsymbol{r}_{\boldsymbol{l}}\mathbf{+}\boldsymbol{r}_{\boldsymbol{s}}\boldsymbol{e}^{\boldsymbol{i\delta}_{\boldsymbol{r}}}}{\mathbf{2}} \right\vert^{\mathbf{2}}$ | (S3) |
|  | $\boldsymbol{R}_{\boldsymbol{cross}}\mathbf{=}\left\vert\boldsymbol{q}_{\boldsymbol{r}} \right\vert^{\mathbf{2}}\mathbf{=}\left\vert\frac{\boldsymbol{r}_{\boldsymbol{l}}\mathbf{-}\boldsymbol{r}_{\boldsymbol{s}}\boldsymbol{e}^{\boldsymbol{i\delta}_{\boldsymbol{r}}}}{\mathbf{2}} \right\vert^{\mathbf{2}}$ | (S4) |

To ensure that the light wave energy is primarily distributed in the transmission space, co-polarized CP and reflected light waves must be suppressed. Specifically, T_cross_ should be maximized, while 𝑇_co_, 𝑅_co_, and R_cross_ should tend toward zero. Numerical simulations of the nanobrick were performed using the CST STUDIO SUITE software. During the simulation, the unit cell boundary conditions were set near the nanostructure, and the orientation angle was set as 0°(normal incidence). A CP plane wave was normally incident on the nanostructure. The cross-polarized and co-polarized transmittances were collected using the transmission field port. The height of the top Si layer was fixed at 355 nm, and the cell size *C* was fixed at 300 nm. The geometric parameters of length *L* and width *W* were swept over the range of 100 to 300 nm with a step size of 10 nm to optimize the performance of the nanobricks. The polarization conversion efficiency of the optimized nanostructure was 64.70% at a design wavelength of 633 nm. Figures S2a and S2b depict the modulation characteristics of a single nanobrick on incident light waves, given structural parameters *L* = 190 nm, *W* = 110  nm, *H* = 355  nm, and *C* = 300  nm. It can be observed that the unit structure exhibits high transmission properties at 633 nm. Figure S2c presents a real photograph of the metasurface sample.

The metasurface sample was fabricated on a fused silica substrate coated with a silicon layer using electron beam lithography (EBL). The nanobrick structures have the following parameters: length *L* = 190  nm, width *W* = 110  nm, height *H* = 355  nm, and periodicity *C* = 300 nm. First, a 355 nm-thick amorphous silicon layer was deposited onto a 500 μm-thick fused silica substrate using plasma-enhanced chemical vapor deposition. Then, an electron beam resist was spin-coated onto the silicon layer, and the desired patterns defined in the GDS file were transferred onto the resist via EBL exposure. Subsequently, a 40 nm-thick chromium (Cr) layer was deposited using an electron-beam evaporator. The sample was then immersed in acetone for lift-off, leaving Cr only in the patterned areas. During the etching process, Cr served as a hard mask, and the exposed regions of the amorphous silicon were selectively removed. After etching, the Cr mask was stripped, leaving behind only the silicon nanobrick array on the fused silica substrate.


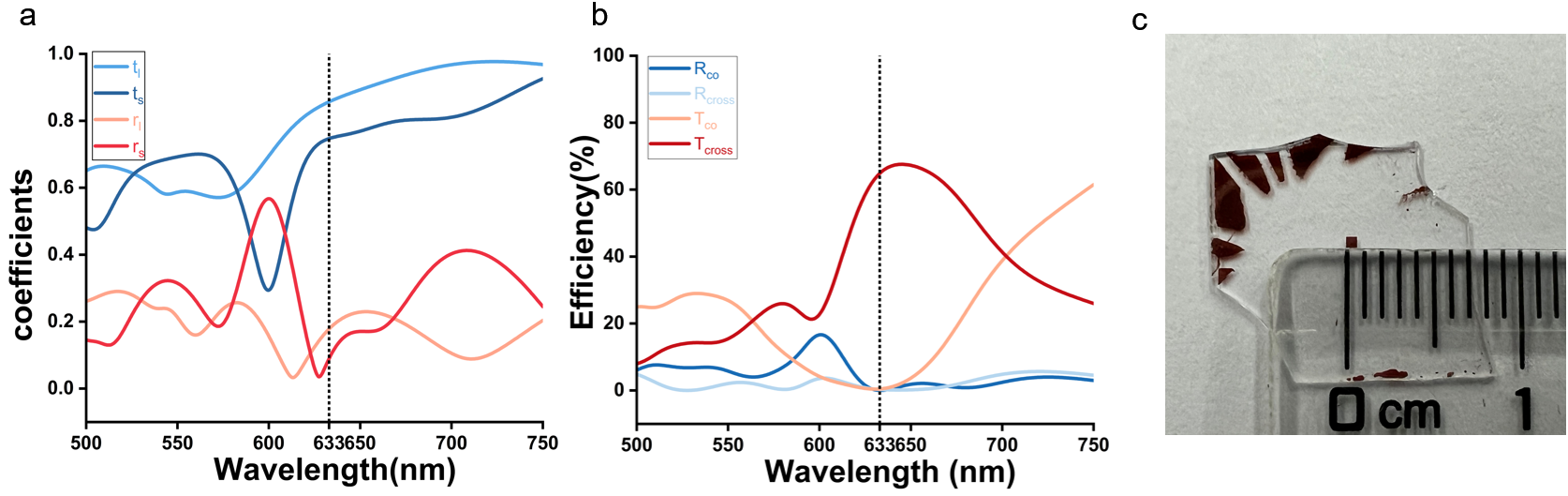


Figure S2. Geometric parameter design of the metasurface unit structure and real sample image. a) Transmission and reflection coefficients as a function of wavelength for linearly polarized incident light along the long and short axes of the nanobrick. b) Modulation efficiencies of transmission and reflection as a function of wavelength. c) Photograph of the metasurface sample.

6. Metasurface Diffraction Efficiency

We conducted a spatial distribution measurement of the diffraction efficiency of the grazing-emission structured light pattern. As shown in Figure S3a, we analyzed the upper half (0°–180° range) of the ring-shaped structured light pattern (red arc) emitted by the metasurface. This semi-circular region was divided into 18 equal angular segments at 10° intervals to evaluate the energy distribution across different directions.In the experiment, we used an optical power meter to measure the structured light energy within each angular segment, thereby obtaining the emission intensity for each direction. The total measured diffraction efficiency was 25.1%.We also performed numerical simulations of the diffraction process in each corresponding region. Figure S3b presents a comparison between the simulated and measured results under an incident laser power of 30 mW. The red curve represents the simulated values, while the blue curve shows the experimental measurements. A noticeable discrepancy exists between the two, primarily due to the following factors:

1. The output beam diameter of the laser is approximately 1.2 mm, which exceeds the effective area of the metasurface, causing part of the energy to remain unmodulated.

2. Pattern deviations and fabrication errors during the nanofabrication process.

3. Additional losses from interface reflections and other optical inefficiencies.


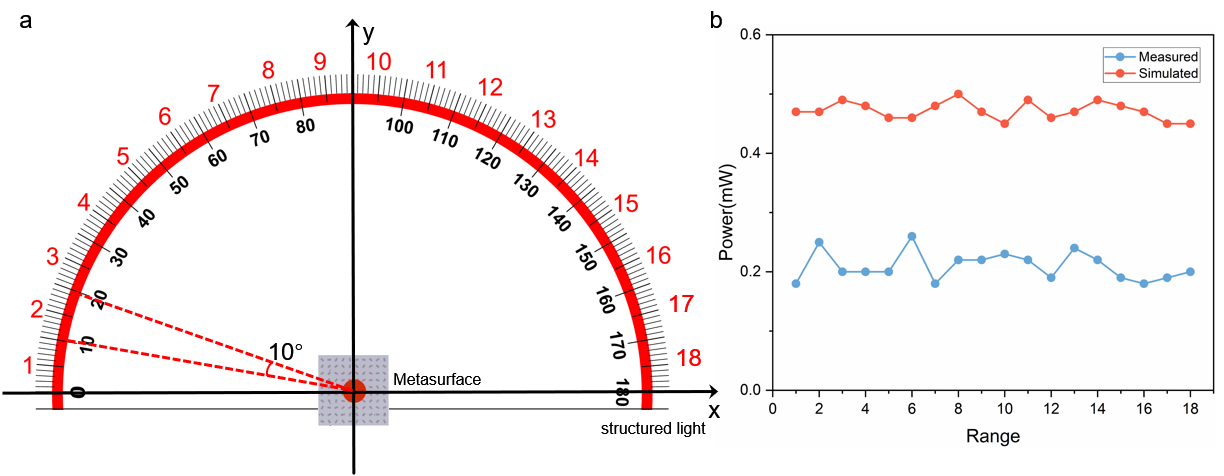


Figure S3. a) Schematic diagram of angular segmentation used for optical power measurements across different regions. b) Comparison between measured and simulated optical power in each region under 30 mW incident light.

7. Reason for Partial Loss of Adaptive Structured Light

The void region observed in the lower part of the structured light pattern in Figure 2(d) is caused by partial obstruction of the grazing-emission beams due to the clamping fixture. Figure S4a presents a schematic of the metasurface under clamping, while Figure S4b shows a front view of the clamped metasurface. As indicated by the yellow-shaded area, the physical presence of the fixture’s base platform prevents certain grazing-angle beams from propagating through, resulting in an incomplete ring projection at the bottom of the structured light pattern.This occlusion is primarily influenced by two experimental factors:

1. Across different experimental setups, minor variations in the vertical distance 𝐻 between the metasurface and the top edge of the clamp arise due to alignment tolerances and fine adjustments of the experimental platform. A larger 𝐻 corresponds to a smaller occlusion area. As a result, the extent of beam cutoff observed from the camera perspective varies accordingly.

2. The left image in Figure 2(d) was captured in a 3-meter-diameter tunnel scenario, while the right image corresponds to a rectangular pipe with a width of 60 cm. These two experiments were conducted in environments with different cross-sectional shapes and sizes, leading to different relative positions and imaging distances between the metasurface and the camera. This shift in relative geometry affects the effective coverage area of the structured light in the captured images and further amplifies the inconsistency of the occluded region.


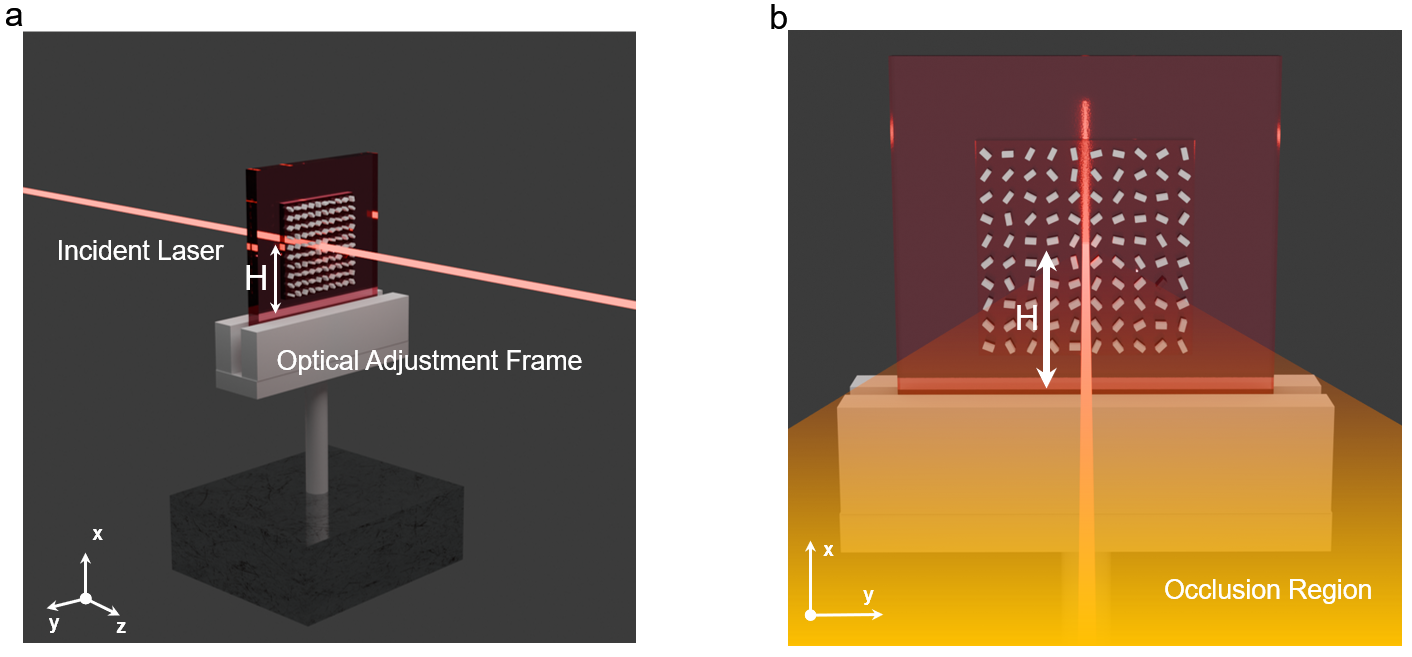


Figure S4. a) Schematic diagram of the metasurface under clamping. b) Front view of the metasurface in the clamped state.

8. Principle of Binocular Ranging

Binocular ranging operates on the disparity method, leveraging two cameras positioned at a fixed distance to observe the same object and determining the object's distance via the position difference (disparity) in the left and right images. Disparity *d* is one of the most fundamental geometric parameters in a stereo vision system. It represents the horizontal pixel offset between the projections of the same 3D point onto the left and right images. In a stereo imaging setup, the two cameras are positioned side by side along a horizontal baseline. Each 3D point in space appears with a slight lateral displacement between the two images—this pixel-level shift is referred to as disparity. The closer an object is to the cameras, the greater the disparity; the farther the object, the smaller the disparity.As illustrated in Figure S5, assume the two cameras of the binocular system are located at points *O*_L_ and *O*_R_, with a baseline length of *B*. The object point *P* is at a distance *Z* from the camera plane, with its projections at *X*_L_ and *X*_R_ on the image planes of the left and right cameras. With the baseline length 𝐵 between the cameras and the focal length 𝑓 are known, the principle of similar triangles yields,

|  | $\frac{\boldsymbol{Z}}{\boldsymbol{f}}\mathbf{=}\frac{\boldsymbol{B}}{\boldsymbol{d}}$ | (S5) |
| --- | --- | --- |
|  | $\boldsymbol{z}\mathbf{=}\frac{\boldsymbol{f}\boldsymbol{\cdot}\boldsymbol{B}}{\boldsymbol{d}}\mathbf{=}\frac{\boldsymbol{f}\boldsymbol{\cdot}\boldsymbol{B}}{\boldsymbol{x}_{\boldsymbol{L}}\mathbf{-}\boldsymbol{x}_{\boldsymbol{R}}}$ | (S6) |


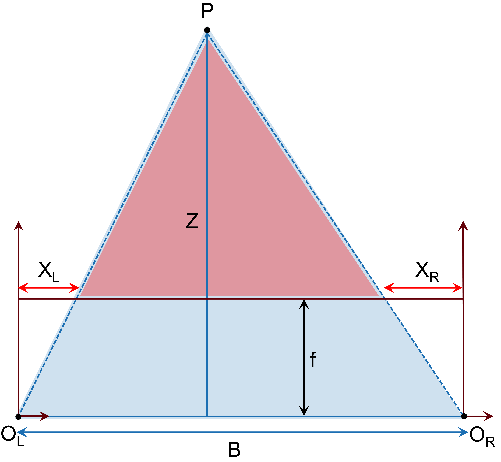


Figure S5. The disparity-based ranging method utilizes the similarity of the red and blue triangles to derive an equation for calculating the distance.

9. Pixel Row Alignment

When the binocular cameras are horizontally aligned on the same plane, the pixel of point 𝑃 in the left image should have its corresponding pixel in the right imagelocated on the same horizontal line or along a corresponding epipolar line. This substantial reduction in computational scope greatly enhances efficiency. The fundamental matrix describes the geometric relationship between two images. ^[2]^ Let the pixel coordinates of point 𝑃 be $\boldsymbol{X}_{\boldsymbol{L}}\boldsymbol{=}\left[ \boldsymbol{x}_{\boldsymbol{L}}\boldsymbol{,}\boldsymbol{y}_{\boldsymbol{L}}\boldsymbol{,1} \right]^{\boldsymbol{T}}$ in the left image and $\boldsymbol{X}_{\boldsymbol{R}}\boldsymbol{=}\left[ \boldsymbol{x}_{\boldsymbol{R}}\boldsymbol{,}\boldsymbol{y}_{\boldsymbol{R}}\boldsymbol{,1} \right]^{\boldsymbol{T}}$ in the right image. The fundamental matrix satisfies the equation,

|  | $\boldsymbol{X}_{\boldsymbol{R}}^{\boldsymbol{T}}\boldsymbol{\cdot}\boldsymbol{F}\boldsymbol{\cdot}\boldsymbol{X}_{\boldsymbol{L}}\mathbf{=0}$ | (S7) |
| --- | --- | --- |

*X*_L_ and *𝑋*_𝑅_ represent the homogeneous coordinates of point 𝑃 in the left and right images, respectively. 𝐹 is the fundamental matrix describing the projection relationship, derived from the camera's intrinsic parameters. To simplify the computation process further, the calculation range should ideally be restricted to the same horizontal line (𝑦_𝐿_ = 𝑦_𝑅_). To achieve this, a transformation matrix 𝐻 is defined as,

|  | $\boldsymbol{H}\mathbf{=}\boldsymbol{R}\mathbf{+}\frac{\boldsymbol{ⅇ\cdot}\boldsymbol{V}^{\boldsymbol{T}}}{\left\Vert\boldsymbol{V} \right\Vert}$ | (S8) |
| --- | --- | --- |

e represents the epipole, the projection of one camera's optical center onto the other camera's image plane, and satisfies 𝐹⋅𝑒 = 0. 𝑅 is a rotation matrix that moves the epipole 𝑒 to the image center. *V* denotes the direction vector of the epipolar line, which is normalized by dividing by its norm to prevent proportional distortion after transformation. By combining a series of rotations and translations, the epipole is moved to the image center, and the epipolar lines are aligned horizontally, significantly enhancing computational efficiency.

10. Calculation of Camera Intrinsic Parameters

Camera calibration is essential for accurate measurements, and in this study, we used Kalibr to perform the calibration. ^[3,4]^ AprilGrid calibration boards, utilizing AprilTag 2D encoding, are commonly used for camera calibration tasks in large-scale and dynamic environments. The method relies on AprilTag's unique encoding and high-contrast design, enabling accurate detection and recognition of feature points using each tag's unique binary code. The AprilGrid board consists of numerous AprilTag markers, each represented as a black-and-white square pattern with a unique binary code, generated by specific algorithms to guarantee the uniqueness and reliability of every tag. Thanks to AprilTag's high recognition accuracy and uniqueness, the system is capable of accurately identifying and localizing each tag, even when partially occluded, offering a reliable basis for the estimation of camera intrinsic and extrinsic parameters. During the calibration process, the spatial coordinates (*X,Y,Z*) of each feature point on the board are predefined. The corresponding points in the image are (*u,v*), determined as pixel coordinates by the detection algorithm. According to the camera imaging model, the relationship between spatial points and image points is expressed by the following formula:

|  | $\boldsymbol{s}\left[ \begin{aligned} \boldsymbol{u} \\ \boldsymbol{v} \\ \mathbf{1} \end{aligned} \right]\mathbf{=}\boldsymbol{K}\left[ \left. \boldsymbol{R} \right\vert\boldsymbol{t} \right]\left[ \begin{aligned} \boldsymbol{X} \\ \boldsymbol{Y} \\ \boldsymbol{Z} \\ \mathbf{1} \end{aligned} \right]$ | (S9) |
| --- | --- | --- |
|  | $\boldsymbol{K}\mathbf{=}\left[ \begin{matrix} \boldsymbol{f}_{\boldsymbol{x}} & \mathbf{0} & \boldsymbol{C}_{\boldsymbol{x}} \\ \mathbf{0} & \boldsymbol{f}_{\boldsymbol{y}} & \boldsymbol{C}_{\boldsymbol{y}} \\ \mathbf{0} & \mathbf{0} & \mathbf{1} \end{matrix} \right]$ | (S10) |

Here, 𝑠 represents the scaling factor used to normalize the projection point coordinates onto the image plane. (*u,v,*1)^T^ denotes the homogeneous coordinates of a feature point in the image coordinate system. *K* is the intrinsic matrix of the camera, with 𝑓_𝑥_ and 𝑓_𝑦_ as the focal lengths along the x and y axes. *C*_𝑥_ and *C*_𝑦_ represent the origin of the image plane coordinate system, corresponding to the projection of the optical axis on the imaging plane, ideally coinciding with the sensor's center. [*R*∣*t*] is the camera extrinsic parameter matrix, which includes the rotation matrix 𝑅 and translation vector 𝑡, describing the relationship between the camera coordinate system and the world coordinate system. To estimate *K* and [*R*∣*t*], multiple feature points in the calibration image need to be simultaneously detected to establish numerous projection relationships. The least squares method is then used to minimize the projection error 𝐸_camera_ of these feature points, thereby obtaining the optimal intrinsic and extrinsic parameters of the camera.

| $\mathbf{E}_{\mathbf{camera}}\mathbf{=}\sum_{\boldsymbol{i}} \left\Vert\left[ \begin{aligned} \boldsymbol{u}_{\boldsymbol{i}} \\ \boldsymbol{v}_{\boldsymbol{i}} \end{aligned} \right]\mathbf{-}\frac{\mathbf{1}}{\boldsymbol{S}}\boldsymbol{k}\left[ \left. \boldsymbol{R} \right\vert\boldsymbol{t} \right]\left[ \begin{aligned} \boldsymbol{X}_{\boldsymbol{ⅈ}} \\ \boldsymbol{Y}_{\boldsymbol{ⅈ}} \\ \boldsymbol{Z}_{\boldsymbol{i}} \\ \mathbf{1} \end{aligned} \right] \right\Vert^{\mathbf{2}}$ | (S11) |
| --- | --- |

11. Stereo Matching Standards

To identify the corresponding pixel of point 𝑃 in the same row of pixels between the left and right camera frames, a matching criterion must be calculated. Normalized Cross-Correlation (NCC) ^[5]^ is a widely adopted similarity metric to assess the similarity of pixel blocks between the left and right images. The formula for NCC is given as:

|  | $\boldsymbol{NCC}\mathbf{=}\frac{\boldsymbol{\Sigma}_{\left( \boldsymbol{i}\mathbf{,}\boldsymbol{j} \right)\boldsymbol{\in}\boldsymbol{W}}\left[ \boldsymbol{I}_{\boldsymbol{L}}\left( \mathbf{ⅈ,}\boldsymbol{j} \right){\boldsymbol{\cdot}\boldsymbol{I}}_{\boldsymbol{R}}\left( \boldsymbol{i}\mathbf{-}\boldsymbol{d}\mathbf{,}\boldsymbol{j} \right) \right]}{\sqrt{\boldsymbol{\Sigma}_{\left( \boldsymbol{i}\mathbf{,}\boldsymbol{j} \right)\boldsymbol{\in}\boldsymbol{W}}\boldsymbol{I}_{\boldsymbol{L}}\left( \mathbf{ⅈ,}\boldsymbol{j} \right)^{\mathbf{2}}\boldsymbol{\cdot}\boldsymbol{\Sigma}_{\left( \boldsymbol{i}\mathbf{,}\boldsymbol{j} \right)\boldsymbol{\in}\boldsymbol{W}}\boldsymbol{I}_{\boldsymbol{R}}\left( \mathbf{ⅈ-}\boldsymbol{d}\mathbf{,}\boldsymbol{j} \right)^{\mathbf{2}}}}$ | (S12) |
| --- | --- | --- |

In this formula, 𝐼_𝐿_(*i,j*) denotes the grayscale value of pixel (*i,j*) in the left image, and 𝐼_𝑅_(*i−d,j*) denotes the grayscale value of pixel in the right image. 𝑊 is the set of matched pixels. The closer the NCC value is to 1, the higher the similarity between the two pixel blocks. To mitigate the impact of dark environments and repetitive features in metasurface light spots, the matching method requires further optimization. Semi-Global Matching (SGM), ^[6]^ a commonly applied optimization algorithm for stereo matching, has the following formula:

|  | $\boldsymbol{E}\left( \boldsymbol{d} \right)\mathbf{=}\sum_{\boldsymbol{p}\boldsymbol{\in}\boldsymbol{P}} \boldsymbol{\{}\boldsymbol{C}\left( \boldsymbol{p}\mathbf{,}\boldsymbol{d}_{\boldsymbol{P}} \right)\mathbf{+}\sum_{\boldsymbol{q}\boldsymbol{\in}\boldsymbol{N}\left( \boldsymbol{p} \right)} {\mathbf{(}\boldsymbol{p}}_{\mathbf{1}}\boldsymbol{\cdot}\left[ \left\vert\boldsymbol{d}_{\boldsymbol{p}}\mathbf{-}\boldsymbol{d}_{\boldsymbol{q}} \right\vert\mathbf{=1} \right]\mathbf{+}\boldsymbol{P}_{\mathbf{2}}\boldsymbol{\cdot}\left[ {\mathbf{\vert}\boldsymbol{d}}_{\boldsymbol{P}}\mathbf{-}\boldsymbol{d}_{\boldsymbol{q}} \right\vert\boldsymbol{>1])\}}$ | (S13) |
| --- | --- | --- |

C(*p*,*d*_P_) represents the NCC calculation value, serving as the initial matching criterion for disparity 𝑑_𝑃_ at pixel 𝑝. If the disparity difference between adjacent pixels 𝑝 and 𝑞 is 1 pixel, a small penalty 𝑃_1_ is applied. For disparity differences greater than 1, a larger penalty 𝑃_2_ is introduced. This mechanism penalizes sharp disparity changes to avoid abrupt transitions in the disparity map. For pixel 𝑝, SGM aggregates costs along multiple paths in different directions, summing them to compute the final cost for pixel

𝑝.

12. Calculation of IMU Intrinsic Parameters

Independent calibration of the IMU is conducted to determine its intrinsic parameters, focusing on the biases and noise characteristics of the accelerometer and gyroscope. More specifically, the accelerometer bias is commonly estimated under stationary conditions. Ideally, the accelerometer should measure only the downward gravitational acceleration (9.81 m/s²) when stationary, but deviations occur due to inherent biases. The accelerometer bias 𝑏_𝑎_ for each axis can be determined by placing the IMU stationary in various orientations, measuring acceleration values, averaging them, and comparing with the known gravitational value. In the same manner, the gyroscope bias

𝑏_g_ is estimated during stationary conditions. Ideally, the angular velocity should be zero when the IMU is stationary, but non-zero outputs indicate bias. Multiple stationary measurements of the gyroscope output are averaged to calculate the bias on each axis. During the calibration process, the IMU is positioned stationary in various orientations, and its outputs are recorded. The derived bias values are used to correct IMU data in real time, removing bias effects and ensuring more accurate acceleration and angular velocity readings, thus enhancing sensor reliability and precision. The bias model is refined during calibration by comparing actual IMU measurements with theoretical values. The calibration error model can be mathematically expressed as:

| $\mathbf{E}_{\mathbf{IMU}}\mathbf{=}\sum_{\boldsymbol{j}} \mathbf{\vert\vert}\boldsymbol{a}_{\boldsymbol{measured}\mathbf{,}\boldsymbol{j}}\mathbf{-}\left( \boldsymbol{R}_{\boldsymbol{\omega,i}}\boldsymbol{\cdot}\boldsymbol{a}_{\boldsymbol{ture}\mathbf{,}\boldsymbol{j}}\mathbf{+}\boldsymbol{b}_{\boldsymbol{a}} \right)\mathbf{\vert\vert}^{\mathbf{2}}\mathbf{+}\sum_{\boldsymbol{j}} \mathbf{\vert\vert}\boldsymbol{\omega}_{\boldsymbol{measured}\mathbf{,}\boldsymbol{j}}\mathbf{-}\left( \boldsymbol{\omega}_{\boldsymbol{ture}\mathbf{,}\boldsymbol{j}}\mathbf{+}\boldsymbol{b}_{\boldsymbol{g}} \right)\mathbf{\vert\vert}^{\mathbf{2}}$ | (S14) |
| --- | --- |

*a*_measured,j_ denotes the acceleration recorded during the 𝑗-th measurement, 𝑎_true,j_ is the theoretical true acceleration, 𝑅_𝜔_,𝑖 is the rotation matrix between the IMU's coordinate system in the 𝑖-th direction and the gravity direction, 𝜔_measured,j_ indicates the angular velocity measured during the 𝑗-th trial, and 𝜔_true,j_ is the theoretical true angular velocity.


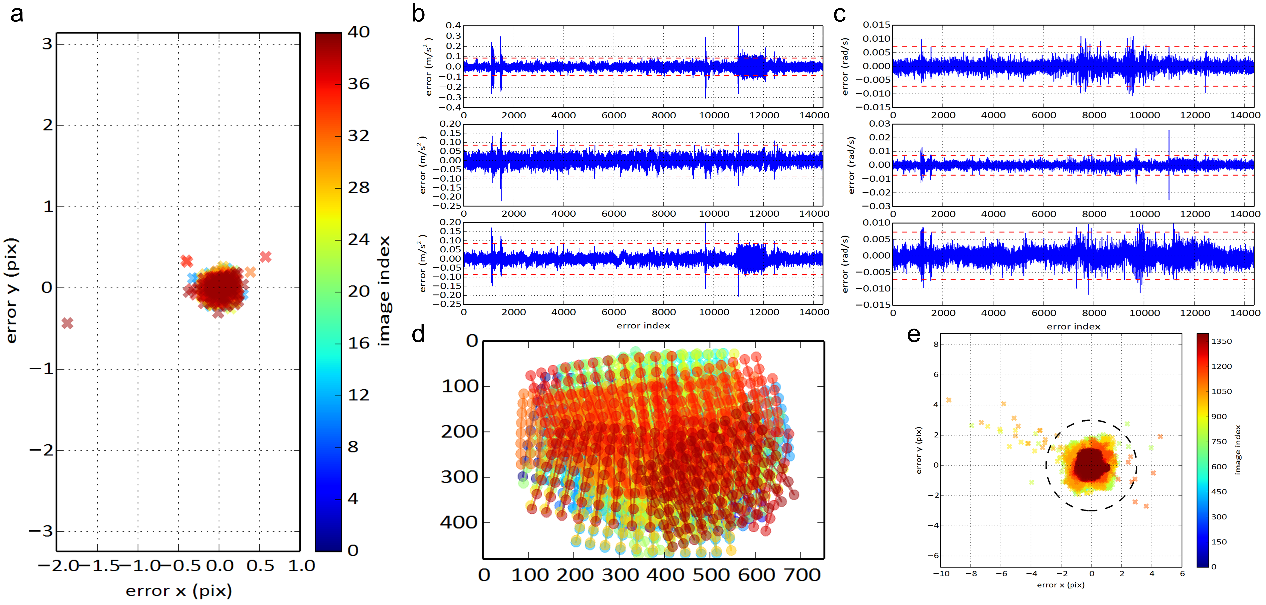


Figure S6. Calibration errors for the binocular camera and IMU system. a) Camera reprojection error, which indicates the discrepancy between the calibrated projection point and the actual point. b) IMU accelerometer error, reflecting the discrepancy between the predicted and actual acceleration values. c) IMU gyroscope error, indicating the difference between predicted and actual angular velocities. d) Feature point distribution in calibration images during the standalone camera calibration process. e) Reprojection error during the joint camera-IMU calibration process, showing the error distribution for the entire system.

13. Joint Calibration of Binocular Camera and IMU

Joint calibration involves multi-sensor data fusion to compute the exact spatial transformation between the camera and IMU, specifically the relative pose extrinsic parameters. Additionally, time synchronization and optimization frameworks ensure consistency between sensor observations, achieving high-precision attitude estimation. Joint calibration demands both spatial and temporal alignment, given that the camera and IMU may operate with differing sampling rates and time shifts. Time synchronization errors are corrected by incorporating a time delay parameter via interpolation and alignment models, forming a time synchronization error term 𝐸_sync_ for optimization of the time differences between the camera and IMU. The total optimization objective function is formulated as:

|  | $\boldsymbol{E}\mathbf{=}\boldsymbol{\lambda}_{\boldsymbol{camera}}\boldsymbol{\cdot}\boldsymbol{E}_{\boldsymbol{camera}}\mathbf{+}\boldsymbol{\lambda}_{\boldsymbol{IMU}}\boldsymbol{\cdot}\boldsymbol{E}_{\boldsymbol{IMU}}\mathbf{+}\boldsymbol{\lambda}_{\boldsymbol{sync}}\boldsymbol{\cdot}\boldsymbol{E}_{\boldsymbol{sync}}$ | (S15) |
| --- | --- | --- |

$\boldsymbol{\lambda}_{\boldsymbol{camera}}$, $\boldsymbol{\lambda}_{\boldsymbol{IMU}}$, and $\boldsymbol{\lambda}_{\boldsymbol{sync}}$ represent weighting factors for balancing visual, inertial, and time synchronization error components. Minimizing this total error function allows for solving the extrinsic matrix between the camera and IMU, the IMU intrinsic biases, and the time synchronization parameters.

14. Pose Estimation Combination

The essential matrix 𝐸 describes the relative motion (relative pose, including rotation and translation) between two adjacent views. ^[7]^ Assume two camera perspectives, where one camera's position has undergone a rotation and translation relative to the other. For a 3D point 𝑃, the projections in the two camera views adhere to the constraints imposed by the essential matrix 𝐸. Given a pair of feature points 𝑃_t_ and 𝑃_t+1_

in two adjacent image frames, the relationship satisfies the following equation:

|  | $\boldsymbol{P}_{\boldsymbol{t}\mathbf{+1}}^{\boldsymbol{T}}\boldsymbol{F}\boldsymbol{P}_{\boldsymbol{t}}\mathbf{=0}$ | (S16) |
| --- | --- | --- |
|  | $\boldsymbol{E}\mathbf{=}\boldsymbol{K}^{\boldsymbol{T}}\boldsymbol{FK}\mathbf{=}\boldsymbol{U\Sigma}\boldsymbol{V}^{\boldsymbol{T}}$ | (S17) |

By leveraging the coordinate relationship of point 𝑝 between adjacent frames, the fundamental matrix 𝐹 can be derived. Along with the camera intrinsic matrix 𝐾, the essential matrix 𝐸 is calculated. To obtain the rotation 𝑅 and translation 𝑡, the essential matrix 𝐸 is subjected to Singular Value Decomposition (SVD), yielding 𝐸 = *U*Σ*V*^T^, where 𝑈 and 𝑉 are orthogonal matrices representing rotation and mapping relationships during the SVD process. *Σ* is a diagonal matrix with its diagonal entries being the singular values. For the essential matrix, two of these singular values are equal, while the third is zero, preserving its unique geometric properties. During the SVD process, 𝑈 and 𝑉's column vectors represent the left and right singular vectors of the essential matrix, respectively. Geometrically, these vectors describe the motion relationship between the two camera coordinate systems, encapsulating both rotation and translation information. To derive the rotation matrix from 𝑈 and 𝑉, a special rotation matrix 𝑊 is introduced, defined as:

|  | $\boldsymbol{W=}\left[ \begin{matrix} \boldsymbol{0} & \boldsymbol{-1} & \boldsymbol{0} \\ \boldsymbol{1} & \boldsymbol{0} & \boldsymbol{0} \\ \boldsymbol{0} & \boldsymbol{0} & \boldsymbol{1} \end{matrix} \right]$ | (S18) |
| --- | --- | --- |

The matrix *W* is an orthogonal matrix that ensures the constructed rotation matrix possesses the correct rotational properties, specifically a determinant of 1. By combining *W*, *U*, and *V*, two candidate rotation matrices can be generated: *R*_1_ = *UWV*^T^ and *R*_2_ = *UW*^T^*V*^T^. The direction of the translation vector *t* is encoded in the third column of the *U* matrix. This arises from the structural characteristics of the essential matrix, which encode the camera's translation information within the singular value decomposition's direction vectors. Specifically, if *U* = [*u*_1_, *u*_2_, *u*_3_], where *u*_3_ is the third column of *U*, then *t* = *u*_3_ can be selected as the direction of the translation vector. Four potential relative pose combinations for the camera are obtained: (*R*_1_, +*t*), (*R*_1_, -*t*), (*R*_2_, +*t*), and (*R*_2_, -*t*). In real-world applications, only one combination is physically meaningful: the 3D reconstructed points must lie in front of the camera (positive depth). We reconstruct the feature points in 3D and determine which combination satisfies the positive depth constraint for all points, ultimately selecting the single valid combination.

15. Measurement Time of 3D Reconstruction

In this study, we primarily validated the system on a slow-moving platform and further examined its dynamic adaptability. The 3D reconstruction process integrates frame-level structured light scanning with stereo matching. The system operates with the following parameters: a camera frame rate of 30 fps, an average image processing time of approximately 33 milliseconds per frame, and an output of about 2,000–3,000 valid spatial points per frame. During experiments, the platform moved at a speed of 0.1 m/s. As a result, the system achieved quasi-real-time frame-by-frame measurement and preliminary point cloud generation, with the reconstruction of a 4-meter-long tunnel segment taking approximately 40 seconds.

Currently, the system supports frame-level real-time 3D reconstruction. The experimental velocity of 0.1 m/s was chosen to match the available computational resources (CPU processing capability) and the image acquisition rate (30 fps). Our experiments confirmed that slower movement imposes no burden on the system and can even improve point cloud sampling density. However, increasing the movement speed poses challenges, as the current platform relies on CPU-based multi-threaded serial execution for image processing and point cloud generation. This leads to risks of data buffering backlog and inter-frame loss.It is important to note that these limitations do not arise from the reconstruction algorithm itself, but rather from constraints in computing resources and data transmission rates. If the system is migrated to a higher-performance platform—such as one with GPU acceleration or embedded heterogeneous processors—and paired with a higher image acquisition rate, it holds the potential to operate in higher-speed scenarios without requiring modification to the core reconstruction method.

16. Comparison with Metasurface-Based LiDAR

Compared with metasurface-based LiDAR systems, ^[8]^ the structured light–vision fusion system proposed in this study differs significantly in system architecture, point cloud density, field-of-view coverage, and applicable scenarios. To clearly highlight the advantages of our system, Table S2 presents a comparison of key performance indicators between the two approaches.The proposed structured light system achieves wide field-of-view and high-density 3D reconstruction without relying on any mechanical scanning components, making it particularly well-suited for measurements in enclosed environments such as tunnels. In contrast, current metasurface-based LiDAR systems still face notable limitations in terms of field-of-view, structural complexity, and cost control. Therefore, our method offers greater feasibility for practical deployment and system-level integration.

Table S2. Comparison Between the Proposed System and Metasurface-Based LiDAR Systems

| Comparison Item | Our | Metasurface-Based LiDAR ^[8]^ | |
| --- | --- | --- | --- |
| Field-of-View Coverage | Metasurface-based omnidirectional grazing emission with FOV up to 176° | | Mostly fixed emission angles; limited FOV; requires additional scanning mechanisms |
| Scanning Components | Fully solid-state | | Often requires rotating structures or acousto-optic deflectors; high complexity |
| System Composition | Camera, laser source, metasurface | | Includes complex optical and electronic modules such as optical switches and frequency comb generators |
| Point Cloud Density | Generates high-density point clouds (>2,000 points per frame) | | Typically sparse; density improved only through repeated scanning |

17. Explanation of Camera Field of View and Relative Position

To ensure that the structured light pattern can be fully captured within a single frame, we made specific design decisions regarding the camera's positioning and field of view, as illustrated in Figure S7.

1. To ensure that both the grazing-emission and normal-emission components of the structured light fall within the camera’s field of view, the camera was placed approximately 80 cm away from the metasurface. At this distance, the camera can effectively cover the entire structured light projection range emitted by the metasurface, including a maximum emission angle of approximately 176°, enabling complete pattern imaging.
2. The current lens selection represents a trade-off between imaging completeness and distortion control. Although fisheye lenses with fields of view greater than 170° can theoretically capture the entire projection range at a much shorter distance, such lenses often introduce severe nonlinear distortions—especially in peripheral areas—leading to geometric compression and blurring. These distortions adversely affect stereo matching and point cloud reconstruction. After extensive testing, we selected an industrial lens with a moderate 150° field of view and low distortion to ensure both stable pattern capture and algorithmic compatibility.

During the experiments, precise central alignment of the metasurface within the tunnel was not required. This is because the designed structured light exhibits a grazing emission diffraction angle of up to 88°, which is sufficient to cover the entire tunnel cross-section. Even under an off-center configuration, the structured light can still reach tunnel wall. However, when positioning the camera, it is important to keep it relatively centered with respect to the tunnel cross-section for two main reasons:

1. To improve measurement efficiency, we aim to capture the complete structured light pattern in a single frame, allowing the 3D reconstruction of one cross-sectional slice without the need for multiple scans or stitching, which can introduce additional errors and time costs. If the camera is offset from the center, portions of the pattern may be occluded or clipped at the frame edges, resulting in degraded point cloud quality and compromised reconstruction performance.
2. To reduce computational load, it is important to avoid capturing regions irrelevant to measurement within the image frame, thus minimizing data processing overhead.

In our experimental setup, the metasurface, laser source, and camera are all mounted on a single platform. Once the camera’s position is properly adjusted, the metasurface naturally aligns to a near-central position as well. We used the live preview window to verify whether the entire structured light pattern was captured, adjusting the camera’s position and orientation accordingly, without relying strictly on geometric center alignment.


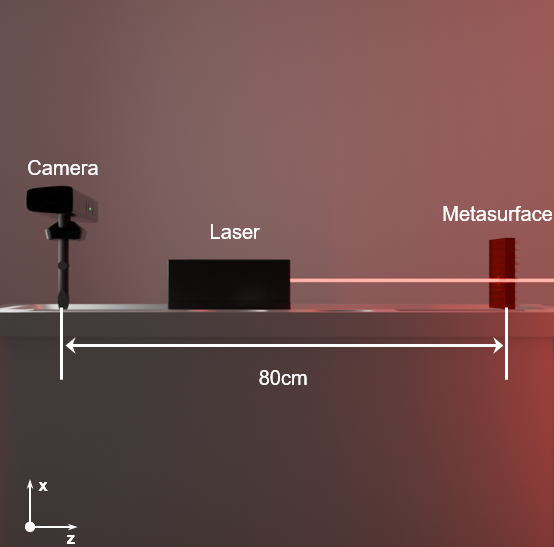


Figure S7. Schematic diagram of the camera position. The camera is placed 80 cm behind the metasurface.

References

[1] Z. Li, Q. Dai, M. Q. Mehmood, G. Hu, B. L. Yanchuk, J. Tao, C. Hao, I. Kim, H. Jeong, G. Zheng, S. Yu, A. Alù, J. Rho, C.-W. Qiu, *Light Sci Appl* 2018, *7*, 63.

[2] J. Rehder, R. Siegwart, P. Furgale, *IEEE Transactions on Robotics* 2016, *32*, 383.

[3] Q. Zhang, R. Pless, in *2004 IEEE/RSJ International Conference on Intelligent Robots and Systems (IROS) (IEEE Cat. No.04CH37566)*, 2004, pp. 2301–2306 vol.3.

[4] R. I. Hartley, *IEEE Transactions on Pattern Analysis and Machine Intelligence* 1997, *19*, 580.

[5] J.-C. Yoo, T. H. Han, *Circuits Syst Signal Process* 2009, *28*, 819.

[6] H. Hirschmuller, in *2005 IEEE Computer Society Conference on Computer Vision and Pattern Recognition (CVPR’05)*, 2005, pp. 807–814 vol. 2.

[7] H. C. Longuet-Higgins, *Nature* 1981, *293*, 133.

[8] N. Li, C. P. Ho, J. Xue, L. W. Lim, G. Chen, Y. H. Fu, L. Y. T. Lee, *Laser Photonics Rev* 2022, 16, 2100511.
